# Electric field control of spin orbit coupling and circular photogalvanic effect in a true ferrielectric crystal

**DOI:** 10.1093/nsr/nwaf320

**Published:** 2025-08-08

**Authors:** Yunlin Lei, Xinyu Yang, Shouyu Wang, Daliang Zhang, Zitao Wang, Jiayou Zhang, Yihao Yang, Chuanshou Wang, Tianqi Xiao, Yinxin Bai, Junjiang Tian, Congcong Chen, Yu Han, Shuai Dong, Junling Wang

**Affiliations:** Department of Physics & Guangdong Provincial Key Laboratory of Functional Oxide Materials and Devices, Southern University of Science and Technology, Shenzhen 518055, China; Key Laboratory of Quantum Materials and Devices of Ministry of Education, School of Physics, Southeast University, Nanjing 211189, China; College of Physics and Materials Science, Tianjin Normal University, Tianjin 300387, China; Multi-scale Porous Materials Center, Institute of Advanced Interdisciplinary Studies & School of Chemistry and Chemical Engineering, Chongqing University, Chongqing 400044, China; State Key Laboratory of Inorganic Synthesis and Preparative Chemistry, Jilin University, Changchun 130012, China; College of Physics and Materials Science, Tianjin Normal University, Tianjin 300387, China; Department of Physics & Guangdong Provincial Key Laboratory of Functional Oxide Materials and Devices, Southern University of Science and Technology, Shenzhen 518055, China; Department of Physics & Guangdong Provincial Key Laboratory of Functional Oxide Materials and Devices, Southern University of Science and Technology, Shenzhen 518055, China; College of Physics and Materials Science, Tianjin Normal University, Tianjin 300387, China; Department of Physics & Guangdong Provincial Key Laboratory of Functional Oxide Materials and Devices, Southern University of Science and Technology, Shenzhen 518055, China; Department of Physics & Guangdong Provincial Key Laboratory of Functional Oxide Materials and Devices, Southern University of Science and Technology, Shenzhen 518055, China; Department of Chemistry, Southern University of Science and Technology, Shenzhen 518055, China; School of Emergent Soft Matter, South China University of Technology, Guangzhou 510640, China; Key Laboratory of Quantum Materials and Devices of Ministry of Education, School of Physics, Southeast University, Nanjing 211189, China; Department of Physics & Guangdong Provincial Key Laboratory of Functional Oxide Materials and Devices, Southern University of Science and Technology, Shenzhen 518055, China; Department of Physics, City University of Hong Kong, Hong Kong, China

**Keywords:** ferrielectric, ferroelectric, circular photogalvanic effect, spin-orbit coupling

## Abstract

Materials possessing long-range ordering of magnetic spins or electric dipoles have been the focus of condensed matter research. Among them, ferri-systems with two sublattices of unequal/non-collinear spins or electric dipoles are expected to combine the properties of ferro- and antiferro-systems, but lack experimental observations in single-phase materials. This is particularly true for the ferrielectric system, since the electric dipoles can usually be redefined to incorporate the two sublattices into one, making it indistinguishable from ferroelectric. This raises doubts about whether or not ferrielectricity can be considered as an independent ferroic order. Here we report the observation of true ferrielectric behaviors in a hybrid single crystal (MV)[SbBr_5_] (MV^2+^ = *N*,*N*′-dimethyl-4,4′-bipyridinium or methyl viologen), where the two electric dipole sublattices switch asynchronously, and thus cannot be reduced to ferroelectric by redefining the unit cell. Furthermore, the complex dipole configuration imparts circularly polarized light sensitivity to the system. An electric field can modulate the non-collinear dipole sublattices and even induce a transition from ferrielectric to ferroelectric state, thereby tuning the helicity-dependent photocurrent. This study opens a new paradigm for the study of true irreducible ferrielectricity (a new class of polar systems) and provides an effective approach to the electric field control of spin-orbit coupling and circular photogalvanic effect.

## Teaser

This study reports the first true irreducible ferrielectric crystal that combines ferroelectric and antiferroelectric features, enabling electric-field control of spinorbit coupling.

## INTRODUCTION

Long-range ordering of magnetic and electric dipoles has given rise to two widely studied families of ferroic materials, and there is an almost one-to-one correspondence between them, i.e. ferromagnetism (FM) vs ferroelectricity, antiferromagnetism vs antiferroelectricity etc. However, ferrimagnetism (FiM), which exhibits an antiparallel arrangement of unequal magnetic dipoles and manifests a net magnetization, e.g. magnetite (Fe_3_O_4_) [1,2], finds fewer counterparts in electric polar systems, except for several reports in liquid crystals [3–5]. Despite being first discussed in the 1960s [6,7], the characteristics of ferrielectricity (FiE) remain elusive. It has been suggested that a ferri-system, FiM or FiE, should reveal the behaviors of both ferro-systems and antiferro-systems macroscopically, i.e. a switchable net magnetization or electric polarization and antiferro–ferro transitions. However, this has not been observed experimentally in single-phase systems, even though it has been reported in composites where ferro- and antiferro- phases coexist [8–11].

In recent years, experimental and theoretical studies have uncovered some potential ferrielectric materials with non-collinearly arranged dipoles, or dipoles of different magnitudes arranged in opposite directions [12–15]. Novel phenomena such as vortex domain structures, negative piezoelectricity and chiral optical responses have been discussed [14,16]. However, their macroscopic behavior, e.g. hysteresis loops, are exactly the same as that of ferroelectric systems (see, for example, CuInP_2_S_6_ [17,18], (MV)[BiI_3_Cl_2_] (MV^2+^=methyl viologen) [19] and BiCu_0.1_Mn_6.9_O_12_ [14]). This is because the polar sublattices in these materials respond to external stimuli simultaneously, and thus can be reduced to one set of dipoles by redefining the unit cell. Phenomenologically, a single-order parameter is sufficient to describe such systems. They are sometimes referred to as ‘reducible’ ferrielectric materials [13]. This raises the question of whether ‘irreducible’ FiE (or true FiE) exists or not, and what would be its distinct properties.

Initial evidence for the ‘irreducible’ FiE in a single-phase material was discovered in BaFe_2_Se_3_ [13,20]. In this compound, the evolution of two sets of electric dipoles upon temperature variation is asynchronous, suggesting that they cannot be reduced to one set of dipoles by doubling the unit cell, and two order parameters would be needed for its phenomenological description. Unfortunately, polarization switching cannot be studied due to its high conductivity.

Here we report the observation of true ‘irreducible’ ferrielectric behaviors in a single-phase hybrid crystal, (MV)[SbBr_5_]. Macroscopic polarization vs electric field (P–E) measurements clearly reveal a switchable net polarization and (two) antiferroelectric–ferroelectric phase transitions. Corresponding switching current (I–E) and small-field capacitance (C–E) measurements confirm the results, which are also corroborated by first principles calculations. The complex dipole arrangement in (MV)[SbBr_5_] imparts chirality to it, leading to circularly polarized light sensitivity that can be tuned by external electric fields. This study opens a new paradigm in the research on hybrid polar materials with exotic dipole arrangements at atomic scale and novel functionalities.

## RESULTS

### Structure and optical properties of (MV)[SbBr_5_]

**Figure 1.**
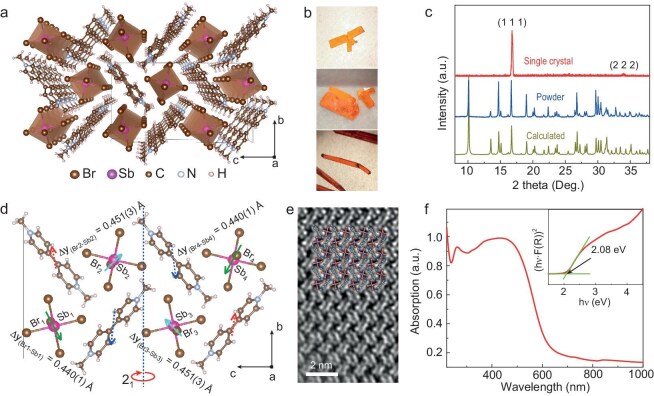
The structure, optical photographs, XRD patterns, TEM image and absorption spectra of (MV)[SbBr_5_]. (a) Schematic illustration of the structure of (MV)[SbBr_5_] with *P*2_1_ space group viewed along the 1D chain. (b) Optical photographs of (MV)[SbBr_5_] single crystals. (c) Powder, single-crystal and calculated XRD patterns of (MV)[SbBr_5_]. (d) The origins of electric dipoles in (MV)[SbBr_5_]. ∆y_(Br__–__Sb)_ denotes the Br–Sb bond length projected along the polar *b*-axis. The arrows represent the magnitudes and directions of the dipole moments generated by the movements of Br relative to Sb and MV cations relative to the inorganic frameworks. (e) Low-dose high-resolution HAADF-STEM image of the (MV)[SbBr_5_] structure along the [100] zone axis, superimposed with the structural model. (f) Absorption spectrum of (MV)[SbBr_5_]. The inset shows the Tauc plot and the estimated direct band gap of 2.08 eV.

We chose the (MV)[MX_5_] family to conduct our search for the ‘irreducible’ FiE based on a previous study that suggests rich polar structures in this hybrid system [21]. The inorganic {MX_5_}_n_ chains along the *a*-axis are packed to generate the framework (Fig. 1a), wherein the MV cations are situated. In this report, we focus on (MV)[SbBr_5_]. The as-prepared single crystals (see Methods for details) show sheet-like, plate-like and rod-like shapes (Fig. 1b), depending on the growth conditions. Single-crystal X-ray diffraction (SC-XRD) and powder X-ray diffraction (PXRD) were employed to verify phase purity and identify the (111) plane of the sheet-like single crystal (Fig. 1c).

**Figure 2.**
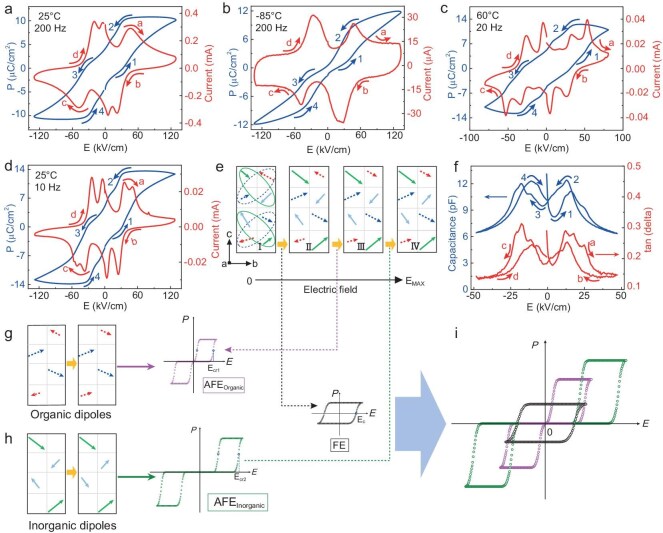
Observation of true ‘irreducible’ ferrielectric behaviors in (MV)[SbBr_5_]. P–E loops and corresponding I–E curves at (a) room temperature (200 Hz), (b) −85°C (200 Hz), (c) 60°C (20 Hz), and (d) room temperature (10 Hz). (e) The proposed polarization reversal pathway under electric field: FiE^(^^−^^)^ → FiE^(+)^ → FE1 → FE2 (Ⅰ → Ⅱ → Ⅲ → Ⅳ); the dashed and solid arrows represent the dipoles contributed by the MV groups and the inorganic frameworks, respectively. (f) Capacitance (blue) and tan δ (red) measured as functions of the DC bias superposed by a small AC driving field (∼3.8 kV/cm) at 800 Hz at room temperature. (g and h) Schematics of AFE–FE transitions of the dipole sublattices from MV groups and inorganic frameworks, respectively, along with the FiE^(^^−^^)^ → FiE^(+)^ loops at low field. (i) Superposition of the three loops leads to the P–E loop of (MV)[SbBr_5_].

A detailed crystallographic analysis at room temperature reveals that the structure of (MV)[SbBr_5_] belongs to the polar space group *P2*_1_, with complex lattice distortions as shown in Fig. 1d. For this compound, Z = 4 and Z′ = 2 (where Z is the number of formula units per unit cell and Z′ is the number of independent formula units in the asymmetric unit). The refined unit cell parameters are a = 6.1042 (6) Å, b = 13.0699 (11) Å, c = 23.5370 (2) Å and β = 94.140 (3)°. Within the unit cell, SbBr_5_ units repeat along the *a*-axis to form a quasi-1D inorganic framework, which is separated by planar MV^2+^ dications inserted between the frameworks. Within the inorganic framework, the Br atoms bridging the Sb atoms (Br_bridging_) along the *a*-axis exhibit positional deviations in the *bc*-plane, oriented towards four different directions and characterized by two different shift magnitudes (indicated by the green and light blue arrows). This configuration results in two sets of electric dipoles from the inorganic chains, leading to the doubling of the unit cell and a complex polar structure. Additionally, a 2_1_ screw axis along the *b*-axis appears, with the electric dipoles associated through the 2_1_-axis being equal on the *bc*-plane. The clear differences in Br–Sb bond lengths (Fig. 2 and Fig. S1) illustrate the distinctions between these two sets of dipoles. To better understand the characteristics of polarization in (MV)[SbBr_5_], we first focus on the dipoles generated by the inorganic framework. By fitting the SC-XRD data, we obtained that Δy_(Br1__–__Sb1)_ = Δy_(Br4__–__Sb4)_ = 0.440 (1) Å ≠ Δy_(Br2__–__Sb2)_ = Δy_(Br3__–__Sb3)_ = 0.451 (3) Å, where Δy_(Br__–__Sb)_ refers to the projection of Br–Sb bond length within the *bc*-plane along the *b*-axis. Furthermore, we also obtained that Δz_(Sb1__–__Sb2)_ = Δz_(Sb3__–__Sb4)_ = 5.869 (1) Å ≠ Δz_(Sb2__–__Sb3)_ = 5.823 (1) Å ≠ Δz_(Sb1__–__Sb4__′__)_ = 5.915 (1) Å, Δy_(Sb1__–__Sb2)_ = 6.528 (3) Å ≠ Δy_(Sb3__–__Sb4)_ = 6.541 (6) Å ≠ Δy_(Sb2__–__Sb3)_ = Δy_(Sb1__–__Sb4__′__)_ = 6.535 (1) Å, where Δz_(Sb__–__Sb)_ and Δy_(Sb__–__Sb)_ refer to the projections of the Sb–Sb distances between neighboring [SbBr_5_] units along the *c*-axis and *b*-axis, respectively. Note that this configuration has a synergistic impact on the MV cations, transforming it from a symmetric arrangement to an asymmetric one (Fig. S2). Furthermore, it prompts uneven arrangements of MV cations along the polar *b*-axis, giving rise to additional electric dipoles. Such intricate configuration endows (MV)[SbBr_5_] with both non-collinear and antiparallel dipoles, while exhibiting a net polarization under zero field. The latter has been confirmed by second harmonic generation (SHG) measurements, as shown in Fig. S3. We have attempted to investigate the relative atomic displacements in (MV)[SbBr_5_] using transmission electron microscopy (TEM). A high-resolution high-angle annular dark-field scanning TEM (HAADF-STEM) image along the [100] zone axis is shown in Fig. 1e and Fig. S4. However, the hybrid crystal is prone to electron beam damage, making only the intercalation of MV groups visible, while the detailed atomic positions within the inorganic framework remain unresolved.

Ultraviolet-visible-near-infrared (UV-Vis-NIR) absorption spectroscopy of (MV)[SbBr_5_] at room temperature (Fig. 1f) reveals strong absorption at around 580 nm. Using the Tauc method [22], we obtained a direct bandgap of ∼2.08 eV, which was further validated by theoretical calculations of the band structure (Fig. S5b). Similar to our previous report on (MV)[SbI_5_], this small bandgap is associated with a flat band contributed by the organic MV cations [23].

### Observation of true ‘irreducible’ ferrielectricity in (MV)[SbBr_5_]

The multiple origins and complex arrangement of the electric dipoles revealed by the crystallographic analysis naturally lead to the question of how the dipoles rotate/reverse under electric fields. To investigate the polarization switching characteristic of (MV)[SbBr_5_], we deposited gold electrodes on the naturally exposed (111) planes (the crystals are too brittle to cut) and measured the P–E hysteresis loops together with the corresponding I–E curves. Immediately, we observe unique features unlike any of the known ferroelectric and/or antiferroelectric systems. As shown in Fig. 2a, at room temperature and 200 Hz, upon increasing the electric field from 0 to ${\mathrm{E}}_{{\mathrm{max}}}^ + $, polarization increases with a corresponding broad peak in the I–E curve. However, when the electric field decreases from ${\mathrm{E}}_{{\mathrm{max}}}^ + $ to 0, two current peaks are observed. Similar features are observed during the 0–${\mathrm{E}}_{{\mathrm{max}}}^ - $–0 cycle. Overall, the P–E loop shows kinks similar to those observed in antiferroelectric materials except a net polarization at zero field (see Supplementary Note S3 for further discussion), but the corresponding I–E curve is very different. Furthermore, when we decrease the temperature to −85°C, only two peaks are observed in the I–E curve during each 0–${{\mathrm{E}}}_{{\mathrm{max}}}$–0 cycle and the P–E loop looks just like that of an antiferroelectric system (Fig. 2b). On the other hand, when the temperature is increased to 60°C and the frequency is reduced to 20 Hz, the broad peak during the 0–${{\mathrm{E}}}_{{\mathrm{max}}}$ scan splits into three, resulting in a total of five peaks in the I–E curve and kinks clearly visible in the P–E loop during each 0–${{\mathrm{E}}}_{{\mathrm{max}}}$–0 cycle (Fig. 2c). Similar behaviors are also observed when we change the measurement frequency, with fewer switching peaks at high frequency and more at low frequency (Fig. 2d and Fig. S6). We also observed the same polarization reversal characteristics by applying electric field along the polar axis (*b*-axis) with asymmetrically arranged electrodes on the (111) surfaces (Fig. S7).

What causes such complex polarization switching characteristics? In general, hybrid ferroelectrics exhibit slower polarization switching dynamics due to the relatively bulky organic groups, which require sufficient time to rotate, translate and/or twist. In contrast, the polarization reversal in inorganic ferroelectrics can occur in a much shorter time. Hence, we speculate that the unique switching characteristics are likely associated with the dipole reversal contributed separately by the movements of the MV groups and the Br ions. Furthermore, since the switching at ∼35 kV/cm is only observed at low frequencies, it likely corresponds to the polarization reversal of the MV groups.

We thus propose a dipole reversal model as schematically shown in Fig. 2e, starting with the initial ferrielectric state [FiE^(^^−^^)^, configuration Ⅰ; the dipole pairs from the inorganic framework (solid arrows) and organic groups (dashed arrows) are marked for clarity]. The corresponding configuration II with opposite net polarization is denoted FiE^(+)^. Under low electric fields, no dipole flipping occurs, but the relative magnitudes of dipoles within each pair reverse, giving rise to the FiE^(^^−^^)^ → FiE^(+)^ hysteresis loop (black) and the small net polarizations at zero field (Fig. 2e, I–II). As electric field increases, antiferroelectric to ferroelectric (AFE–FE, Fig. 2e, II–IV) phase transitions occur, but with different critical fields for the organic (E_cr1_, pink) and inorganic (E_cr2_, green) sublattices. In fact, at a low temperature of −85°C, this transition may not even occur for the organic sublattice, thus a single double hysteresis loop is observed in Fig. 2b and Fig. S8. As temperature increases and/or measurement frequency lowers, the ferrielectric sublattices of both the organic and inorganic origins are activated. Essentially, we observe two antiferroelectric hysteresis loops (each produces two current peaks during 0–${{\mathrm{E}}}_{{\mathrm{max}}}$–0 scan in the I–E curve) added together, resulting in the macroscopic P–E loop and I–E curves shown in Fig. 2c and d, and schematically illustrated in Fig. 2g, h and i. By adjusting parameters of the FiE^(−)^ → FiE^(+)^ loop and the two antiferroelectric loops, we can qualitatively reproduce the P–E loops observed under different conditions (Fig. S9).

The complex electric dipole switching process is also reflected in the small signal C–E response of the single crystal (Fig. 2f). At an AC frequency of 800 Hz with DC bias sweeping from −48 to 48 kV/cm at room temperature. The C–E curve shows three peaks in this process, similar to the I–E curve measured at high temperature and low frequency (Fig. 2c). This is because the small signal C–E measurement is highly sensitive to the instabilities of the dipole sublattices, and the DC sweeping is conducted very slowly. Under relatively small electric fields, the initial FiE state becomes unstable, and subsequent polarization reversal of the net polarization occurs. With the increase of the DC electric field, the ferrielectric states contributed by MV cations and the inorganic framework destabilize sequentially, leading to AFE–FE transitions and two more peaks in the C–E curve.

### First principles calculations

**Figure 3.**
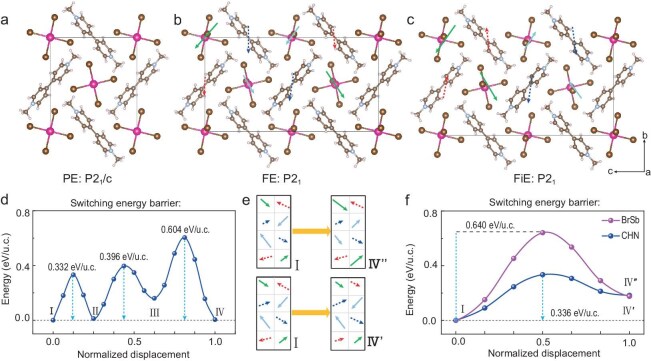
Energy barriers for dipole reversal in (MV)[SbBr_5_]. Schematic illustrations of the (a) non-polar *P*2_1_/*c* paraelectric (PE) phase, (b) polar *P*2_1_ ferroelectric (FE) phase and (c) polar *P*2_1_ ferrielectric (FiE) phase. (d) Energy barriers of the dipole reversal process shown in Fig. 2e. (e) Schematic diagrams illustrating the dipole reversal contributed by the inorganic frameworks and the movement of MV cations during the FiE^(^^−^^)^ → FE (Ⅰ → Ⅳ). (f) FiE^(^^−^^)^ → FE involves the polarization reversal energy barrier associated with the inorganic framework (purple line: Ⅰ → Ⅳ″) and with the movement of MV cations (blue line: Ⅰ → Ⅳ′).

To corroborate the model we proposed, especially that the dipole sublattices from the inorganic and organic components switch independently, we conducted first principles calculations (Supplementary Note S5) to extract the energy barriers for the different dipole reversals. Note that when a large electric field is applied along the *b*-axis, (MV)[SbBr_5_] evolves into a ferroelectric phase with the *P*2_1_ space group (Fig. 3b). On the other hand, when the electric field is along the *a*-axis, it evolves into a ferroelectric phase with the *Pc* space group (Fig. S10c). We thus computed the energies for four distinct dipole configurations of (MV)[SbBr_5_], revealing that the paraelectric (Fig. 3a), *P2*_1_ and *Pc* phases possess energies that are 720.8, 5.2 and 6.4 meV per unit cell (u.c.) higher than the ferrielectric phase (Fig. 3c), respectively. During our experiments, the electric field is applied along the [111] direction due to restrictions of crystal facets. Therefore both *P2*_1_ and *Pc* are possible final phases. We have attempted to determine the final phase by *in*  *situ* Raman spectra under a DC electric field, but the samples broke down under prolonged application of the DC field necessary for Raman measurements. However, since the polarization component of the *P2*_1_ phase along [111] obtained from density functional theory (DFT) calculations (17.81 μC/cm², Table S3) is much closer to the experimental value (about 14 μC/cm²) compared to that of the *Pc* phase (39.92 μC/cm², Table S3), the high-field ferroelectric phase is considered to belong to the *P2*_1_ space group. Why this phase is favored in our measurements requires further study, but it doesn't affect our conclusions here.

To obtain the dipole reversal barriers, we start with the initial ferrielectric state [FiE^(−)^]. The energy barrier for Ⅰ–Ⅱ transition (Fig. 3d) is 0.332 eV/u.c., much lower than that for a direct transition from FiE^(^^−^^)^ to FE (0.872 eV/u.c., Fig. S11b). Notably, if we independently consider the dipole reversals associated with the inorganic framework and MV groups during the FiE^(^^−^^)^ → FiE^(+)^ → FE1 → FE2 processes, we find that the barriers for MV-related dipoles are significantly lower than those for inorganic framework-related dipoles. For example, a direct FiE^(^^−^^)^ → FE1 transition, where FE1 represents a state in which only the organic sublattice has undergone the AFE–FE transition (Ⅳ′ in Fig. 3e), experiences an energy barrier of 0.336 eV/u.c  (Fig. 3f). On the other hand, a direct FiE^(^^−^^)^ → FE2 transition, where FE2 represents the state in which only the inorganic sublattice has undergone the AFE–FE transition (Ⅳ″ in Fig. 3e), experiences an energy barrier of 0.640 eV/u.c (Fig. 3f). This indicates distinct critical fields for the two processes, consistent with our earlier analysis.

### Circular photogalvanic effect in (MV)[SbBr_5_]

**Figure 4.**
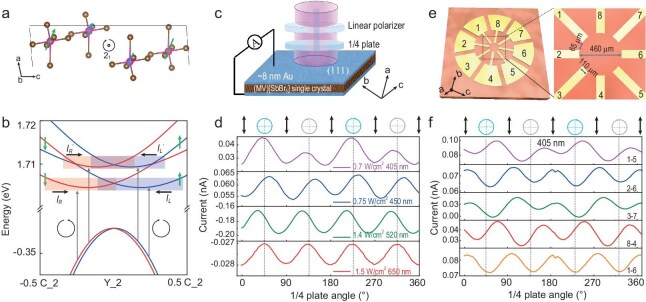
Circular photogalvanic effect in (MV)[SbBr_5_]. (a) Top view of the inorganic sublattice showing the 2_1_-screw axes perpendicular to the inorganic chains. (b) Spin-polarized band structure of (MV)[SbBr_5_] along the C_2–Y_2 direction with SOC considered. (c) The experimental setup to measure the linear and circular photogalvanic effects. (d) The room-temperature photocurrent in (MV)[SbBr_5_] crystal under lights of different wavelengths vs quarter-wave (λ/4) plate rotation angle. (e) Schematic diagram of the device for the measurement of the anisotropic CPGE effect. (f) Photocurrent vs λ/4 plate rotation angle for electrodes with different numbers.

While the first principles calculations corroborate our model of the electric dipoles switching process in (MV)[SbBr_5_], the complex dipole structure also brings about unique functionalities. For example, the non-collinear and unequal Br shifts within the *bc*-plane not only lead to a net polarization and linear photogalvanic effect (LPGE), but also a 2_1_-screw axis along the *b*-axis and a helical arrangement of dipoles within the *ac*-plane, showing chirality (Fig. 4a). This implies different responses to circularly polarized light and possibly electric field tunability. Furthermore, in semiconductors that lack inversion centers, spin-orbit coupling (SOC) splits the spin-degenerate bands, resulting in the Rashba–Dresselhaus effect [24,25]. The two Rashba bands have opposite spins, so light excites spin-up carriers preferably for a given helicity, spin-down for the opposite helicity [26–28], giving rise to circular photogalvanic effect (CPGE) [29,30]. (MV)[SbBr_5_], with the presence of the heavy element Sb and the chiral dipole configuration, offers an ideal platform for studying circularly polarized light response. On the one hand, low electric fields may change the angle between the non-collinear dipoles, thus modulating the SOC. On the other hand, large electric fields induce FiE–FE transitions, which is expected to significantly increase the net polarization and the SOC. Both can be revealed by *in*  *situ* CPGE measurements.

First principles calculations indeed confirm the spin polarized band structure schematically shown in Fig. 4b (Fig. S14). The spin splitting of the valence band primarily occurs perpendicular to the polarization direction, while the spin splitting of the conduction band mainly occurs along the polarization direction. It is important to note that in (MV)[SbBr_5_], the conduction band minimum is mainly determined by the organic cation MV (Fig. S5), with a minor contribution from the inorganic framework, resulting in the absence of significant spin splitting (Fig. S14c). To observe CPGE, higher-energy photon excitation would be required.

Plate-shape single crystals of (MV)[SbBr_5_] (about 100 μm thick) similar to those used in the ferrielectric characterizations were coated with approximately 8 nm of gold electrodes on the (111) surfaces for photoelectric measurements. Light was vertically incident on the (111) surface, and half-wave and quarter-wave plates were used to modulate the light polarization (Fig. 4c). Cosine oscillations of photocurrent with the polarization direction of linearly polarized light were observed under lights of four different wavelengths, demonstrating the presence of linearly polarized light-sensitive bulk photovoltaic effect, i.e. LPGE under zero field (Fig. S15). A reversal of photocurrent direction (for the same electric polarization direction) when the light wavelength goes below 520 nm indicates that under 520 and 650 nm light excitation, electrons are excited to the conduction band contributed by MV cations, whereas under 405 and 450 nm light excitation, electrons are excited to the higher band contributed by the inorganic framework. This is consistent with that observed in (MV)[SbI_5_] [23]. As shown in Fig. 4d, when a quarter-wave plate is used, there is no difference in photocurrent when going from right-handed (RCP) to left-handed (LCP) circular polarization under 520 and 650 nm light excitation, while under 405 and 450 nm light excitation, a significant CPGE signal was detected (differences between photocurrents under LCP and RCP lights). This is consistent with the fact that the conduction band contributed by MV cations does not exhibit spin splitting (Fig. S14c), resulting in photocurrent independent of the helicity of circularly polarized light. On the other hand, the higher band contributed by the inorganic framework exhibits significant spin splitting, thus giving rise to uneven numbers of electrons with opposite momenta when excited by RCP or LCP light.

A set of in-plane electrodes as shown schematically in Fig. 4e were prepared to investigate the correlation between photocurrent and the spontaneous polarization of (MV)[SbBr_5_]. As shown in Fig. 4f, the photocurrent helicity sensitivity, i.e. the photocurrent difference between RCP and LCP illumination, reverses between measurements along 1–5 and 3–7 electrode pairs. This is consistent with the fact that spin splitting occurs in the valence band along the *a*-axis and conduction band along the *b*-axis, respectively, which leads to opposite helicity-dependent photocurrents (Fig. S16). When measured along the 1–6 electrode pair, the photocurrents under RCP and LCP become nearly identical because the effects of spin splitting along the *a*-axis and *b*-axis nearly cancel out and there is no spin splitting along the *c*-axis.

The phenomenological description of light polarization-dependent photocurrent can be as follows [31,27]:


(1) \begin{eqnarray*}
{I}_{pc} &=& C\sin 2\varphi + L\sin \left( {4\varphi + {\varphi }_0} \right)\\
&&+ D\cos \left( {4\varphi + {\varphi }_0} \right) + A,
\end{eqnarray*}

where *C, L, D* and *A* represent the amplitudes of circular photocurrent, the linear bulk photovoltaic current, the linear photon drag effect current caused by momentum transfer from photons to electrons [32] and the offset caused by other effects such as photothermal effects etc., respectively. ${\varphi }_0$ is to compensate for some misalignments in optics. Under zero bias (Fig. S17), the values of *C, L, D* and *A* are 9.52, 10.6, 0.8 and 298 pA, respectively.

**Figure 5.**
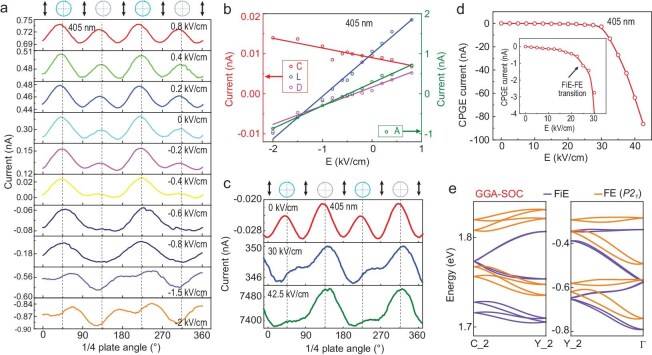
Circular photogalvanic effect in (MV)[SbBr_5_] under low and high electric fields. (a) Photocurrent versus the λ/4 plate angle under different external bias. (b) The different contributions to the photocurrent. (c) Photocurrent versus the λ/4 plate angle under low and high bias. (d) The external electric field dependence of CPGE photocurrent; inset is a magnified view between 0 and 30 kV/cm. (e) Comparison of the bands between the ground-state FiE and the electric-field-induced ferroelectric state (*P*2_1_ phase) considering SOC.

When subjected to low external electric fields, the photocurrents reveal clear changes, as shown in Fig. 5a. Under 405 nm laser irradiation, a change in the direction of current occurs below −0.4 kV/cm of external bias (close to the open circuit voltage of −4 V, Fig. S18b), and the shape of the current–light polarization dependence curve also changes. Above −0.4 kV/cm, the RCP photocurrent is greater than the LCP photocurrent, while below −0.4 kV/cm, the RCP photocurrent is smaller than the LCP photocurrent. Figure 5b shows the variation of the four parameters *C, L, D* and *A* fitted according to Eq. (1) under different external electric fields (see Supplementary Note S7 for detailed discussion). It should be noted that under different fields, the direction of the current due to circular bulk photogalvanic effect (BPGE) remains unchanged. Under short-circuit conditions (*E *= 0), the red and blue square regions in Fig. 4b can be occupied by electrons excited by RCP and LCP light, respectively. Under bias, external bias can change the distribution of available state density in *k*-space, thereby changing the magnitudes of $I_{ph}^R$ and $I_{ph}^L$ [26,28].

The change in CPGE under low fields can be understood if we consider that the dipole sublattices and the net polarization are changed by the external field, which then affect the spin splitting of the bands, as shown in Fig. S20a. Increasing polarization causes the points −k_1_ and k_2_ to shift along −k_y_, resulting in an increase in the net photocurrent *I*_L_. Consequently, the CPGE photocurrent coefficient C = 2|*I*_L_| also increases. Conversely, when a bias opposite to the polarization direction is applied, the polarization decreases, and C decreases as well. With ΔC representing the compensation of the CPGE photocurrent coefficient due to the change in polarization under bias, the CPGE photocurrent coefficient C_exp_ should decrease with increasing field, which is consistent with the experimental observations. A detailed discussion on the microscopic mechanism of linear photon drag effect and the changes in the photocurrent under an external bias is presented in Supplementary Note S8 [33,34].

The above analysis implies that, with higher fields inducing a transition from the ground-state FiE to the ferroelectric state in (MV)[SbBr_5_], a significant increase in polarization will further strengthen the SOC, and a substantial impact on the CPGE current should be expected. We thus prepared thinner (approximately 40 μm thick) plate-shaped (MV)[SbBr_5_] single crystals and repeated the measurements. As shown in Fig. S23, the current–electric field curve measured reproduces the polarization reversal characteristics of the thicker samples. By applying an electric field of 0 → 50 → 0 kV/cm, the thin (MV)[SbBr_5_] crystal was driven into a single-domain state. Subsequently, 405 nm light was vertically irradiated onto the (111) surface of (MV)[SbBr_5_], and a DC field was applied to measure the current under this illumination. As shown in Fig. 5c, under increasing external bias, the shape of the photocurrent underwent significant changes. Additionally, both the magnitude of the photocurrent and the difference between LCP and RCP light illumination are dramatically altered. The helicity-dependent photocurrent ‘*C*’ as a function of the external electric field is shown in Fig. 5d. As the electric field increases, *C* slowly increases at first, then rises sharply around 25 kV/cm, corresponding to the current peak at 25 kV/cm in the current–electric field curve (Fig. S23), which is associated with the ‘FiE–FE’ transition, suggesting a significantly enhanced SOC in the electric field-induced FE phase.

As shown in Fig. 5e and Fig. S24, first principle calculations confirm that the spin splitting in the valence bands along the ‘Γ–A’ and ‘Y_2–Γ’ paths, as well as in the conduction bands along the ‘A–E’ and ‘C_2–Y_2’ paths, are significantly enhanced when the system transitions from the FiE state to the *P2*_1_ FE state. When the excited electrons are primarily governed by spin splitting in the conduction band, as shown in Fig. S25a, under LCP light, the current generated by photoelectrons excited at −*k_1_* increases during the relaxation process, while that excited at *k_2_* decreases, leading to an overall increase in the net photocurrent. The same is true when the excited electrons are governed by the valence band as well (Fig. S25b).

## CONCLUSIONS

In summary, we report the observation of true ‘irreducible’ ferrielectric behaviors in a hybrid crystal (MV)[SbBr_5_] with complex non-collinear dipole arrangements. Temperature- and frequency-dependent measurements indicate that the electric dipoles from the MV groups and the inorganic framework in (MV)[SbBr_5_] undergo asynchronous switching, leading to the combination of a ferroelectric hysteresis loop and (two) AFE–FE transitions. First principles calculations confirm the model by revealing that the dipoles of MV groups have a smaller switching barrier. The complex non-collinear dipole arrangement also confers chirality to (MV)[SbBr_5_]. We observed that the photocurrent depends on the circularly polarized light helicity and achieved modulation of helicity-dependent photocurrent through electric field. This helicity-dependent photocurrent modulation is fundamentally driven by the electric field's influence on the non-collinear dipole sublattices and the FiE–FE transition, which enhances polarization and, in turn, strengthens the SOC. This true ferrielectric order, distinct from both ferroelectric and antiferroelectric orders, offers a new paradigm for the study of ferroic systems and provides a novel approach for the electric field control of SOC and circular photogalvanic effect.

## Supplementary Material

nwaf320_Supplementary_data
