# Supplementary material for: Electric field control of spin orbit coupling and circular photogalvanic effect in a true ferrielectric crystal
Source: Natl Sci Rev. 2025 Aug 8;12(9):nwaf320. doi: 10.1093/nsr/nwaf320 (PMC12421575; doi:10.1093/nsr/nwaf320)
Supplement: nwaf320_Supplementary_data [file nwaf320_supplementary_data.pdf]

Supplementary Information for

Electric Field Control of Spin Orbit Coupling and Circular Photogalvanic Effect in a True Ferrielectric Crystal

Yunlin Lei[1#], Xinyu Yang[2#], Shouyu Wang[3], Daliang Zhang[4], Zitao Wang[6], Jiayou Zhang[3], Yihao Yang[1], Chuanshou Wang[1], Tianqi Xiao[3], Yinxin Bai[1], Junjiang Tian[1], Congcong Chen[8], Yu Han[7], Shuai Dong[2*], Junling Wang[5, 1*]

[1] Department of Physics & Guangdong Provincial Key Laboratory of Functional Oxide Materials and Devices, Southern University of Science and Technology, Shenzhen 518055, Guangdong, China

[2] Key Laboratory of Quantum Materials and Devices of Ministry of Education, School of Physics, Southeast University, Nanjing 211189, China

[3] College of physics and Materials Science, Tianjin Normal University, Tianjin 300387, China

[4] Multi-scale Porous Materials Center, Institute of Advanced Interdisciplinary Studies & School of Chemistry and Chemical Engineering, Chongqing University, Chongqing 400044, China

[5] Department of Physics, City University of Hong Kong, Kowloon 999077, Hong Kong SAR, China

[6] State Key Laboratory of Inorganic Synthesis and Preparative Chemistry, Jilin University, Changchun 130012, China

[7] School of Emergent Soft Matter, South China University of Technology, Guangzhou 510640, China

[8] Department of Chemistry, Southern University of Science and Technology Shenzhen, Guangdong 518055, China

*Corresponding authors: sdong@seu.edu.cn and j.wang@cityu.edu.hk

#: These authors contributed equally to this work.

Note S1: Materials and Methods

Materials: Antimony tribromide ($SbBr_3$, Alfa Aesar), 4,4′-bipyridine (Mreda), Hydrobromic acid (HBr, 48%, Macklin), Methanol (MeOH, Xilong scientific). All the chemicals were bought and used without further purification.

Synthesis of (MV)[$SbBr_5$]: The (MV)[$SbBr_5$] were synthesized by using solvothermal reaction method with bipyridine radical cations molecular and metal halide compound. Firstly, 0.12 g of $SbBr_3$, 0.8 ~ 0.9 mL of HBr (48%), 0.052 g of 4,4′-bipyridine, and 10 mL MeOH were mixed in a 25 mL Teflon bomb. The Teflon bomb was then sealed in a Parr autoclave and heated in a programmable oven with the following parameters: heating from 25 °C to the designated temperature of 150 °C, holding at 150 °C for 30 h, and then cooling to 25 °C with 2 ~ 4 °C/h. This process yielded mostly rod-shaped crystals with a smaller amount of sheet- and plate-shaped crystals. A larger quantity of HBr and a faster cooling rate led to more sheet-shaped crystals.

Structural characterizations: Variable-temperature single-crystal diffraction data of (MV)[$SbBr_5$] were collected on a Rigaku XtalAB PRO MM007DW diffractometer with Mo-Kα radiation (λ = 0.77 Å) at 200 K and room-temperature, respectively. Data were collected and reduced using the Bruker APEX3 program. The structures were determined by a direct method and refined with the OLEX2 program package based on F^2 with refinements of full-matrix least squares. For the structure at

room temperature, R_1:3.2%, wR_2:7.81%, Goof:1.09. Powder X-ray diffraction (PXRD) for (MV)[$SbBr_5$] was performed on a Rigaku D-max 2500 PC with Cu-Kα radiation at room temperature. Ultralow-dose high-resolution transmission electron microscopy (HRTEM) was conducted using a double-Cs-corrected Spectra 300 transmission electron microscope, operated at an acceleration voltage of 300 kV. The HRTEM images were acquired with a Gatan K3 direct-detection camera, utilizing electron-counting mode to maximize detection efficiency. To minimize the structural damage, the electron dose was meticulously regulated to remain < 20 e/$Å^2$. Furthermore, an averaged background filter was employed to augment the signal-to-noise ratio, thereby enhancing image quality. Ultraviolet-visible-near-infrared (UV-vis-NIR) absorption spectra measurement was performed at room temperature using a UV-3600 UV-vis-NIR spectrophotometer.

Electrical characterizations: For ferroelectric measurements, gold electrodes of approximately 15 nm thick were deposited onto the (111) planes of plate-shaped crystals using magnetron sputtering. The polarization-electric field (P-E) hysteresis loops, current-electric field curves and capacitance-electric field (C-E) response were measured by TF Analyzer 3000. For temperature dependent measurements, the samples were cooled using liquid nitrogen, with temperature control facilitated by the TCU 3016. Photoelectric measurements were performed with a parallel electrode configuration. The light source consisted of laser diodes (KYD405NX-T1685, KYD450N100-T1685, KYD520N100-G2290 and D650NX-T1685, Shenzhen Jukun Optical Technology Co., Ltd, China.) emitting light at wavelengths of 405 nm, 450

nm, 520 nm and 650 nm, respectively. The photocurrent response under different laser illuminations were measured using Keithley 2636B. For the gold electrodes grown on the (111) plane to measure the photocurrent in Fig. 4d, Fig. 5a, and Fig. 5c, the areas of the gold electrodes are 1 mm^2, 6.2 mm^2, and 1.8 mm^2, respectively.

Density functional theory (DFT) calculations: The density functional theory (DFT) calculations were carried out using projector-augmented wave pseudopotentials as implemented in the Vienna *ab initio* Simulation Package (VASP) [1]. The exchange-correlation functional was treated using Perdew-Burke-Ernzerhof (PBE) parametrization of the generalized gradient approximation (GGA) [2]. More tests with different exchange-correlation functionals (PBEsol) [3] can be found in Table S2, in comparison with the experimental ones. The van der Waals (vdW) correction of the DFT-D3 method was applied [4]. The energy cutoff was fixed to 500 eV and the k-point grids of 6×3×2 were adopted for both optimization and static calculation. The convergent criterion for the energy was set to 10^{-6} eV, and the criterion of the Hellman-Feynman force during the structural relaxation was 0.01 eV/Å for all atoms. To obtain more accurate band gaps, the hybrid Heyd-Scuseria-Ernzerhof (HSE06) functional was employed, and the energy convergent criterion was set to 10^{-4} eV for the HSE06 functional [5]. The most likely switching pathways among different transition states were evaluated using the nudged elastic band (NEB) method [6]. The theoretical value of ferrielectric polarization was estimated to be 0.29 μC/cm^2 along the *b*-axis based on the standard Berry phase method [7]. Additionally, we have estimated the local dipole moments of every

(MV)[$SbBr_5$] pair (A, B, C and D) along the *b*-axis, as illustrated in Fig. S10. Every pair contain one $[MV]^{2+}$ molecule and one $[SbBr_5]^{2-}$ group. As summarized in Table S3. The net dipole of one unit cell after compensation is 0.38 |e|Å along the *b*-axis (corresponding to P_b=0.33 μC/cm^2, very close to aforementioned net polarization).

Note S2: Relative polarization vectors of the inorganic and organic dipoles.

To provide a clearer microscopic understanding of the origin of the net polarization, we adopted an approximate approach to list the dipole moment vectors of the inorganic and organic groups in Table S1. For the contribution of the $SbBr_5$ units, we approximate the dipole moment vector along the direction of the Br_{bridge}-Sb bond. For the MV^{2+} organic cation, the dipole moment is defined by assigning the center of positive charge to the midpoint between the two nitrogen atoms, and the center of negative charge to the midpoint between the two adjacent Sb atoms within the same $SbBr_5$ framework. This vectorial assignment captures the relative orientation of the organic dipoles. The individual MV dipole vectors are labeled from left to right as ①, ②, ③, and ④ in Fig. S1. This method provides an intuitive geometric picture of how the local dipoles—both inorganic and organic—are aligned to produce the net polarization observed in the structure.

Note S3: Further analysis of the remnant polarization.

There is a substantial remnant polarization after the application of an electric field under ambient condition, as shown in Fig. 2a and 2d. This is attributed to the

close energy proximity between the ferroelectric and ferrielectric phases, as confirmed in the DFT section. By increasing the measurement frequency, reducing the driving electric field, or lowering the temperature, the remnant polarization at zero field decreases (Fig. 2b and S6), approaching the calculated net polarization value (~0.33 $\mu C/cm^2$ along *b*-axis) of the ground ferrielectric phase. This indicates that after the application of the electric field results in a transition from ferrielectric to ferroelectric phases, the structure can revert to the ferrielectric phase once the electric field is removed, though this recovery may require some time due to the proximity of their energies.

Note S4: Characterization of polarization reversal features under an electric field applied along the b-axis.

We applied asymmetric electrodes using silver paste on the two (111) surfaces of the plate-like (MV)[$SbBr_5$] crystals, with the connection between the two electrodes approximately along the b-axis, as shown in Fig. S7a. At 35°C, we observed P-E loops with features similar to those observed when the electric field was applied along the [111] direction. We also observed the same frequency dependence as shown in Fig. S7 b-e. After performing 30 consecutive P-E loop measurements at the same frequency, the shape of the P-E loops remained unchanged, indicating the recovery of the ground FiE state after the AFE-FE transition.

Note S5: DFT and further analysis of the polarization reversal pathways.

By rotating the dipoles in the ground-state FiE phase and optimizing the structure using DFT, we obtained two possible ferroelectric phases with $P2_1$ and Pc space groups corresponding to when the electric field is applied along *b*-axis and *a*-axis respectively, as shown in Fig. S10b and S10c. To evaluate the polarization of the local dipoles, we define one MV^{2+} molecule in conjunction with one $[SbBr_5]^{2-}$ group as forming an uncharged pair. The local dipole moments of each (MV)$[SbBr_5]$ pair (A, B, C, and D) are estimated along *a*, *b* and *c*-axis, as illustrated in Fig. S10. As summarized in Table S3, the total dipoles per unit cell are calculated to be (0, 0.33, 0), (0, 43.73, 0), and (-18.63, 0, -81.35) $\mu C/cm^2$ for the FiE phase, the $P2_1$ FE phase, and the Pc FE phase , respectively. These values are close to values obtained using the standard Berry phase method, which were computed as (0, 0.29, 0), (0, 43.96, 0), and (-18.48, 0, -81.64) $\mu C/cm^2$. The polarization values of four (MV)$[SbBr_5]$ pairs of the intermediate phase, corresponding to the AFE-FE transition occurring only in the organic part, have also been calculated and listed in Table S3. However, since the AFE-FE transition in the organic part is expected to influence the inorganic part, these values may differ slightly from the actual ones. Furthermore, since the experimentally observed polarization value when the electric field is applied along [111] direction is close to that of the $P2_1$ FE phase when projected onto [111] direction (Table S3), we conclude that $P2_1$ FE phase is the high field FE phase in our study.

As shown in Fig. S12, when the electric field is applied along the *a*-axis, the dipoles that are initially aligned opposite to the electric field gradually orient in the direction of the field. First, the AFE-FE transition of the organic dipoles occurs,

followed by the AFE-FE transition of the inorganic dipoles. Meanwhile, the net polarization disappears along the *b*-axis and appears along both the *a*-axis and *c*-axis.

Note S6: Helicity-sensitive photocurrent.

As shown in Fig. S18a, under the 405nm laser irradiation, the photocurrent under RCP light is significantly higher than that under LCP light. The anisotropy factor (g_{Iph}) is calculated by the following equation: $g_{Iph} = 2(I_{ph}^{R} - I_{ph}^{L})/(I_{ph}^{R} + I_{ph}^{L})$, I_{ph}^{R} and I_{ph}^{R} are the photocurrents under RCP and LCP lights, respectively. The measured value of g_{Iph} under 405 nm laser irradiation is 0.064.

Note S7: The microscopic mechanism behind the change in the CPGE photocurrent.

We seek to understand the microscopic mechanism of CPGE to explain the changes of the photocurrent under external bias. For CPGE, the process is enabled by converting the angular momentum of photons into the translational motion of charge carriers [8]. As shown in Supplementary Fig. 19a, under excitation by RCP/LCP light at a frequency of ω, energy and momentum conservation permit electron transitions at two different k_y values. During the relaxation of photo-excited electrons, the asymmetry momentum distribution of electrons results in a net short-circuit currents I_R or I_L. RCP/LCP light have opposite angular momenta, thus the CPGE currents generated by RCP/LCP light have the same magnitude but opposite directions. As shown in Supplementary Fig. 19b and Supplementary Fig. 19c, assuming the bias

does not alter the intrinsic band structure of the material and neglecting spin splitting in the valence band, and C_0 denote the CPGE photocurrent coefficient under an external bias. Under an external bias, the excited electrons gain additional momentum, altering their distribution in the conduction band. This causes the photocurrents I_R and I_L, generated under RCP/LCP light excitation, to increase or decrease by equal amounts. Since the CPGE coefficient C_0 is defined as half the difference between the photocurrents under right and left circularly polarized light, i.e., $C_0 = (I_R - I_L)/2$, we expect C_0 to not change sign, consistent with our experimental observations.

Note S8: The microscopic mechanism behind the change in the LPDE photocurrent under an external electric field.

Regarding the effect of bias on LPDE, LPDE is generated by the linear momentum transfer from photons to charge carriers [8, 9]. For simplicity, we ignore SOC-bands in the LPDE analysis (Fig. S21 a-d). As shown in Fig. S21a, photons carrying in-plane momentum (indicated by tilted dashed arrows) can create non-uniform momentum distribution (from k_3 to k_4) of photoexcited electrons in the conduction band, with net momentum marked by red rectangles. Under bias, the distribution of excited electrons changes, which affects the net momentum. At higher negative bias, this results in a sign change of the LPDE photocurrent, aligning with our experimental observations. Although we have provided a physical explanation for CPGE and LPDE under bias, the linear photogalvanic effect under bias requires and awaits a more comprehensive theoretical treatment.

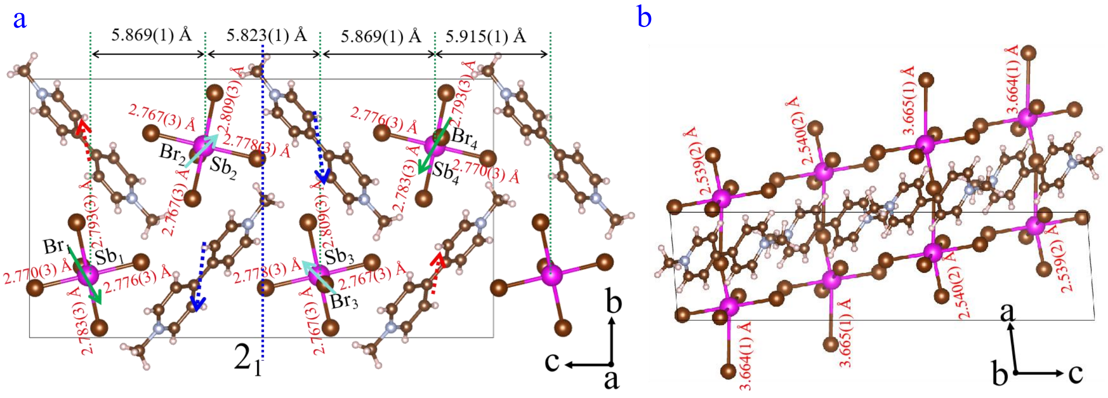

Fig. S1. Bond lengths within the inorganic framework of (MV)[$SbBr_5$]. a, The distances between the inorganic frameworks and the organic groups along the *c*-axis and the lengths of the lateral Br-Sb bonds. b, The lengths of the axial Br-Sb bonds in the inorganic framework.

Table S1. Relative polarization vectors of the inorganic and organic dipoles.

Inorganic dipoles		Organic dipoles	
Br_1-Sb_1	(0.40259, -0.03366, -0.01345)	①	(-0.0042, 0.50011, 0.00213)
Br_2-Sb_2	(0.40264, 0.03453, -0.01311)	②	(0.0055, -0.50338, 0.00113)
Br_3-Sb_3	(-0.40264, 0.03453, 0.01311)	③	(-0.0055, 0.49662, -0.00113)
Br_4-Sb_4	(-0.40259, -0.03366, 0.01345)	④	(0.0042, -0.49989, -0.00213)

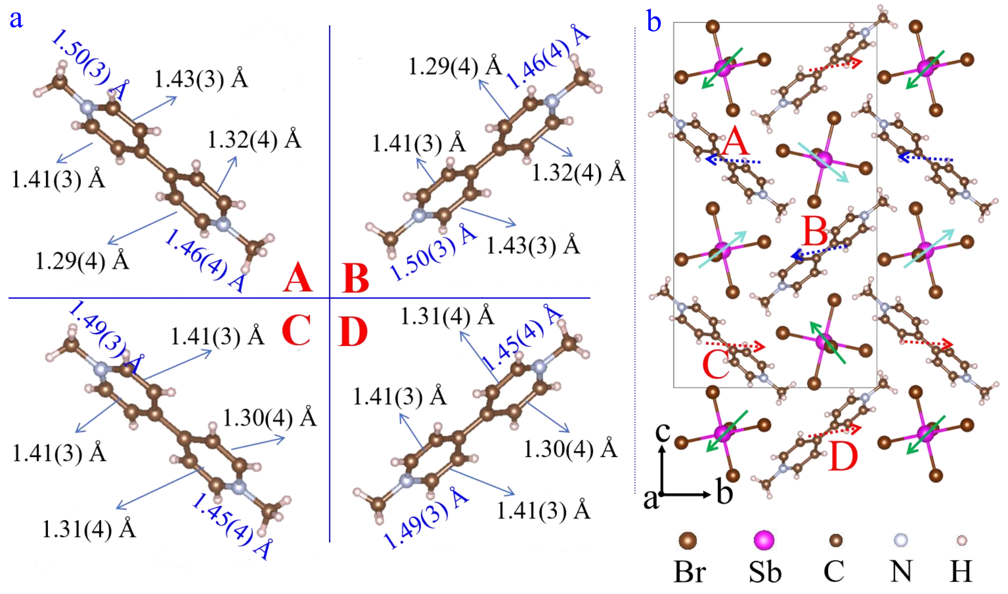

Fig. S2. Distortions of the MV groups in (MV)[$SbBr_5$]. a, The various bond lengths in the methylviologen (MV) cations, indicating the transition from a symmetric structure to an asymmetric structure. b, The locations of the MV groups in the crystal.

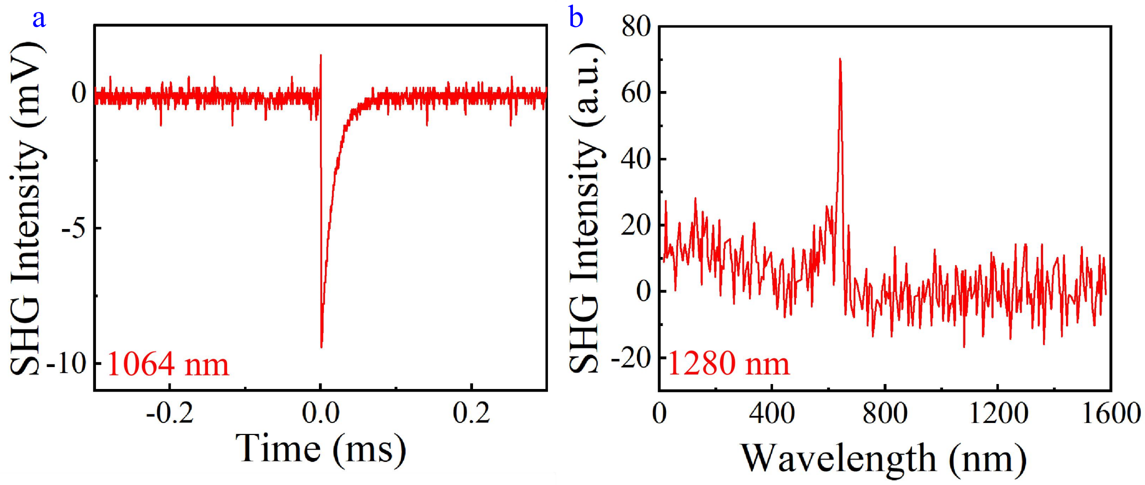

Fig. S3. The SHG of the (MV)[$SbBr_5$] crystal. a, The SHG signals of (MV)[$SbBr_5$] were measured by the Kurtz-Perry method on a Q-switched Nd:YAG solid-state laser with 1064 nm at room temperature, crystals were ground and sieved into a particlesize range 244−355 μm. b, SHG spectra of (MV)[$SbBr_5$] crystal under 1280 nm laser irradiation.

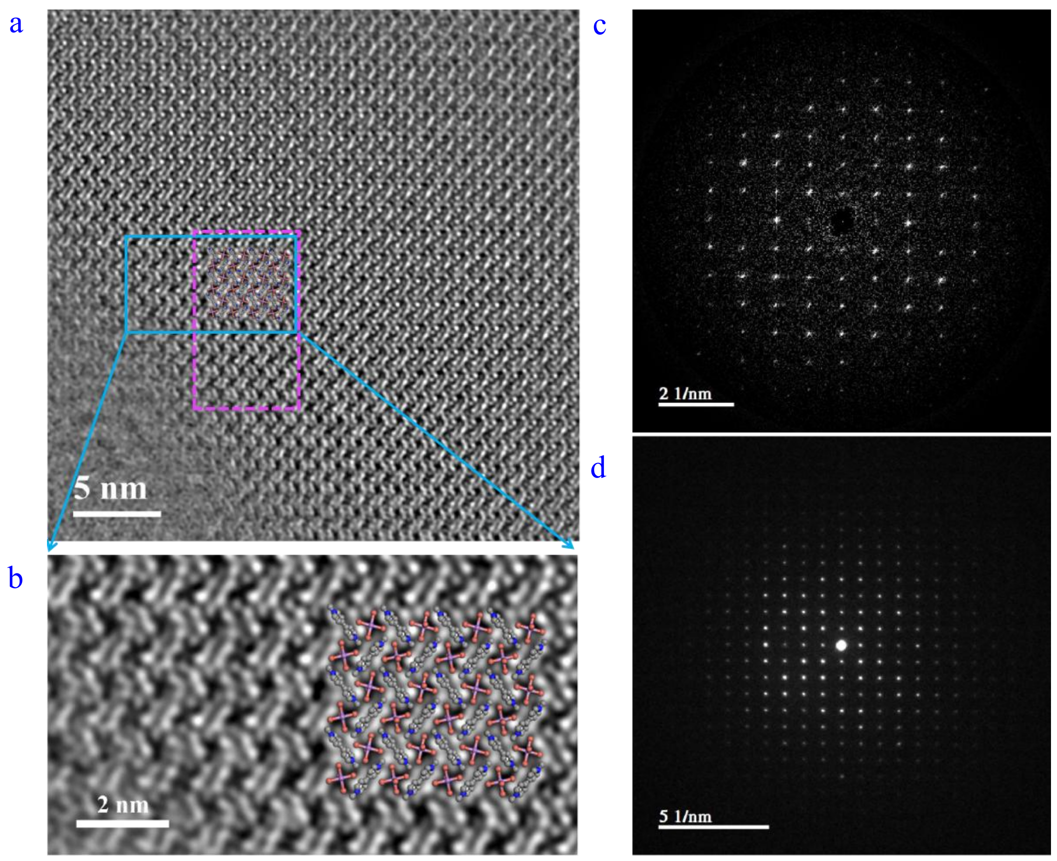

Fig. S4. TEM Characterizations of the (MV)[$SbBr_5$]. a, Low-dose high resolution TEM image of (MV)[$SbBr_5$] structure along the [1 0 0] zone axis, The magnified view of the region outlined by the pink dashed box is shown in the Fig. 1e; b, The magnified image of low-dose high resolution TEM image of the (MV)[$SbBr_5$], superimposed with the structural model. Fast Fourier Transform (FFT) of the image (c) and SAED simulations (d) of (MV)[$SbBr_5$].

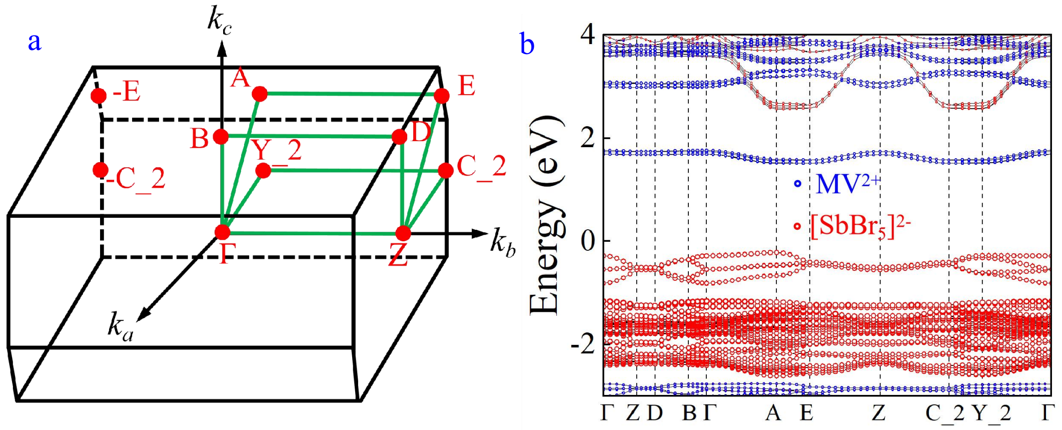

Fig. S5. Density functional theory (DFT) calculations. a, The Brillouin zone of (MV)[$SbBr_5$]; b, DFT-HSE band structure of (MV)[$SbBr_5$], which leads to a band gap ~1.73 eV.

Table S2 The structural parameters of (MV)[$SbBr_5$] at 200 K and 290 K, compared with the DFT (GGA) calculated values.

	Space group	a (Å)	b (Å)	c (Å)	α = γ	β	V (Å^3)
200 K	$P2_1$	6.076	12.905	23.666	90°	94.035°	1851.106
290 K	$P2_1$	6.104	13.070	23.537	90°	94.140°	1872.912
GGA-PBE-D3	$P2_1$	6.191	12.768	23.460	90°	94.691°	1848.174
GGA-PBEsol	$P2_1$	6.034	12.763	24.324	90°	95.743°	1863.976
GGA-PBEsol-D3	$P2_1$	5.969	12.371	23.425	90°	94.162°	1725.164

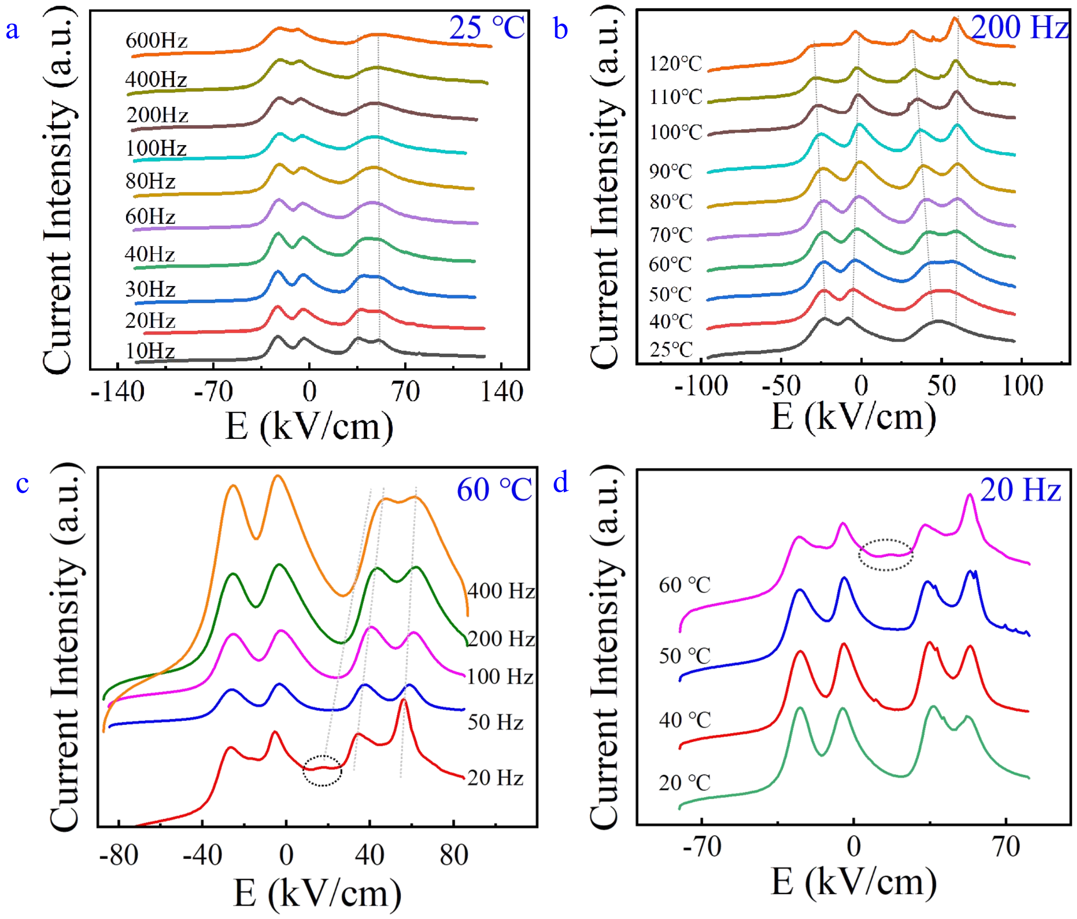

Fig. S6. Variation in the number of switching current peaks as functions of temperature and frequency. a, Variation in the number of current peaks in the first and second quadrants of current-electric field curves at different frequencies. b, Variation in current peaks at 200 Hz frequency and different temperatures, c, Variation in current peaks at 60 °C and different frequencies. d, Variation in current peaks at 20 Hz frequency and different temperatures.

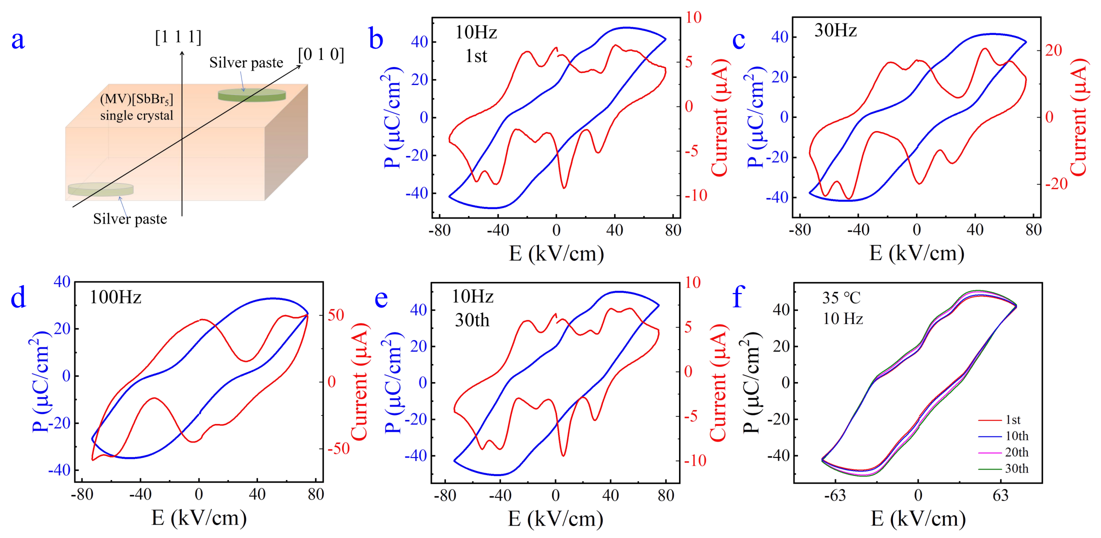

Fig. S7. Characterization of polarization reversal behavior along the b-axis (polar axis). a, Schematic diagram of polarization reversal characterization along the b-axis, where silver paste is applied to two asymmetric positions on the upper and lower (111)-plane of the (MV)$[SbBr_5]$ single crystal, and the line connecting the two electrodes is approximately along the b-axis. P-E loops and corresponding I-E curves at 35 °C measured at (b) 10 Hz, (c) 30 Hz and (d) 100 Hz. e, The P-E loop and corresponding I-E curve after 30 consecutive tests at 35 °C and a frequency of 10 Hz. F, Comparison of the P-E loops during the first, 10th, 20th, and 30th tests at 35 °C and a frequency of 10 Hz.

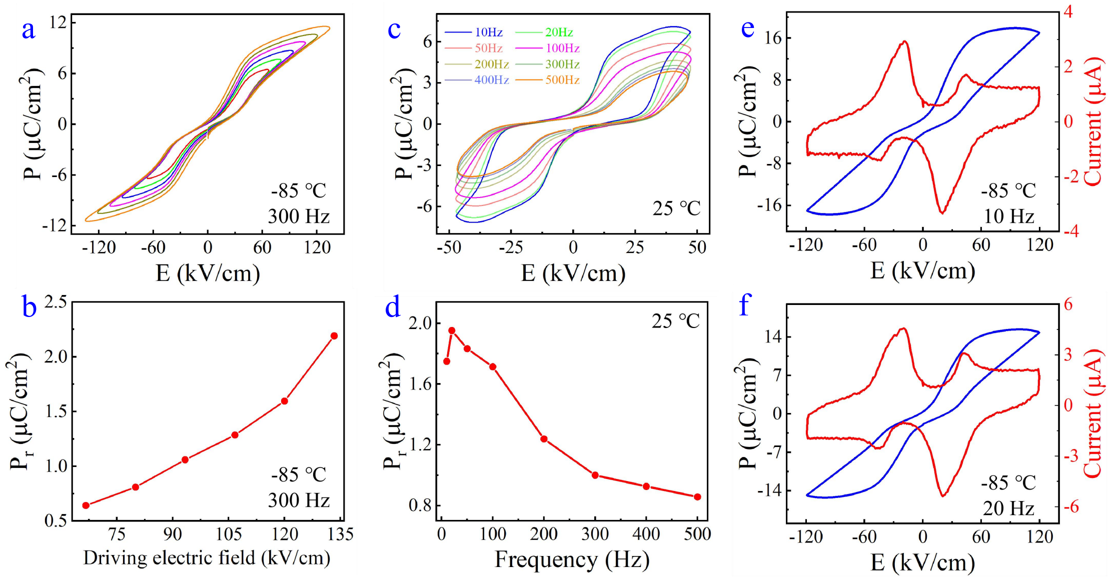

Fig. S8. Further analysis of remnant polarization. a, b, Hysteresis loops measured at -85 °C and 300 Hz under different driving electric fields and the corresponding dependence of remnant polarization along [111] on the driving electric field. c, d, Hysteresis loops measured at room temperature under low driving electric fields at different frequencies and the corresponding dependence of remnant polarization along [111] on frequency. e, f, P-E loops and corresponding I-E curves at -85 °C measured at 200 Hz.

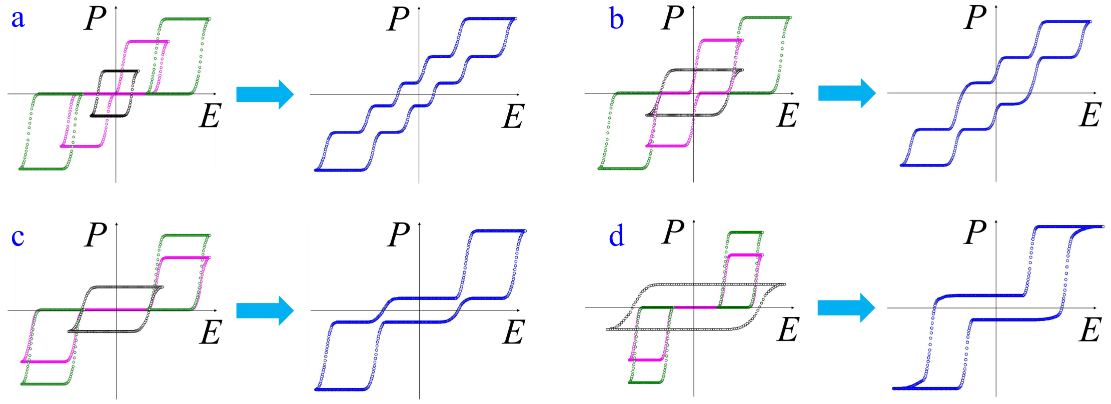

Fig. S9. Schematics of the hysteresis loop obtained with different parameters of the dipole reversal process. The numbers of current peaks during the 0 to E_{max} and E_{max} to 0 sweepings are denoted *m* and *n*, respectively. The coercive field of the FE loop (black) is denoted E_c, and the critical fields for the AFE-FE transitions of the MV groups and the inorganic sublattices are denoted E_{cr1} and E_{cr2}, respectively. a, $E_c < E_{cr1} < E_{cr2}$, m = 3 , n = 2; b, $E_c \approx E_{cr1} < E_{cr2}$, m = 2 , n = 2; c, $E_c < E_{cr1} \approx E_{cr2}$, m = 2, n = 1; d, $E_c \approx E_{cr1} \approx E_{cr2}$, m = 1, n = 1;

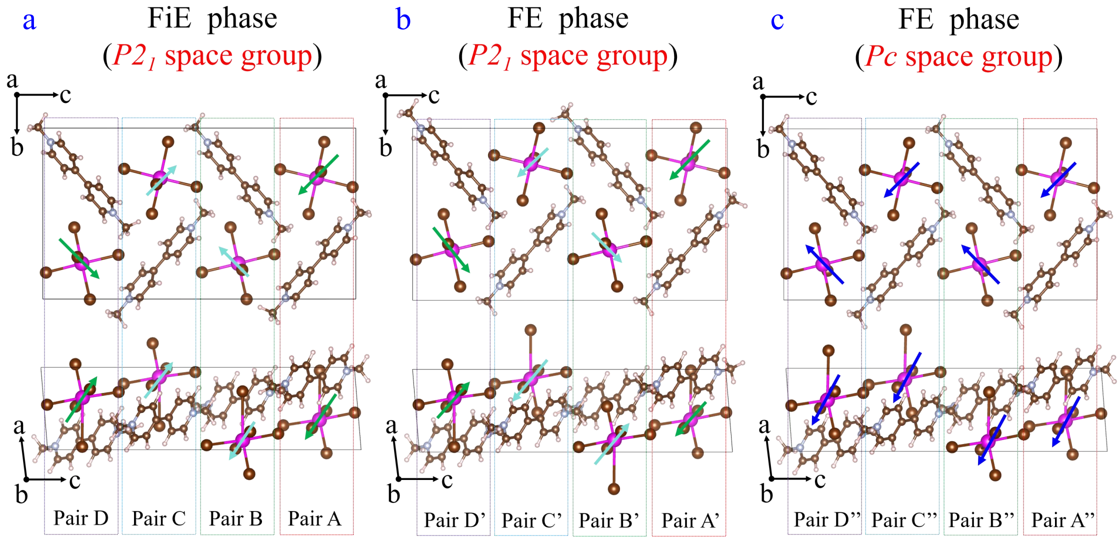

Fig. S10. Schematic illustrations of every pairs (A/A'/A'', B/B'/B'', C/C'/C'' and D/D'/D'') for the ferrielectric phase with $P2_1$ space group (a), the ferroelectric phase with $P2_1$ space group (b), and the ferroelectric phase with *Pc* space group (c). The arrows represent the magnitudes and directions of the dipole moments generated by the movements of Br relative to Sb.

Table S3. The calculated local dipole moments of each (MV)[$SbBr_5$] pairs for the ferrielectric and ferroelectric phases.

Dipole moment (|e|Å)	FiE (II) $P2_1$ space group	III	FE (IV) $P2_1$ space group	③	FE (④) *Pc* space group
Pair A/A'/A''	(0.79, -0.05, 0.04)	(-4.95,12.84, -23.40)	(-5.28,12.68, -23.46)	(-5.61, 12.74, -23.44)	(-5.38, 12.58, -23.47)
Pair B/B'/B''	(0.91, 0.24, -0.01)	(1.13, 0.12, 0.03)	(5.29,12.56, 23.47)	(-5.48, -12.61, -23.47)	(-5.37,-12.58, -23.46)
Pair C/C'/C''	(-0.91, 0.24, 0.01)	(-0.53, 0.22, 0.04)	(-5.29, 12.56, -23.47)	(-1.3, 0.14, -0.01)	(-5.38, 12.58, -23.47)
Pair D/D'/D''	(-0.79, -0.05, -0.04)	(5.57, 12.82, 23.47)	(5.28, 12.68, 23.46)	(-1.17, 0.00, -0.04)	(-5.37, -12.58, -23.46)
Total Dipole (A+B+C+D) (|e|Å)	(0, 0.38, 0)	(1.22, 26, 0.14)	(0, 50.48, 0)	(-13.56, 0.27, -46.96)	(-21.50, 0, -93.86)
Total Dipole (μC/cm^2)	(0, 0.33, 0)	(1.06, 22.52, 0.12)	(0, 43.73, 0)	(-11.75, 0.23, -40.70)	(-18.63, 0, -81.35)
Berry phase-P (μC/cm^2)	(0, 0.29, 0)	(1.16, 22.35, 0.37)	(0, 43.96,0)	(-11.89, 0.17, -40.74)	(-18.48, 0, -81.64)
[1 1 1]-P (μC/cm^2)	0.12	10.19	17.81	-22.20	-39.92

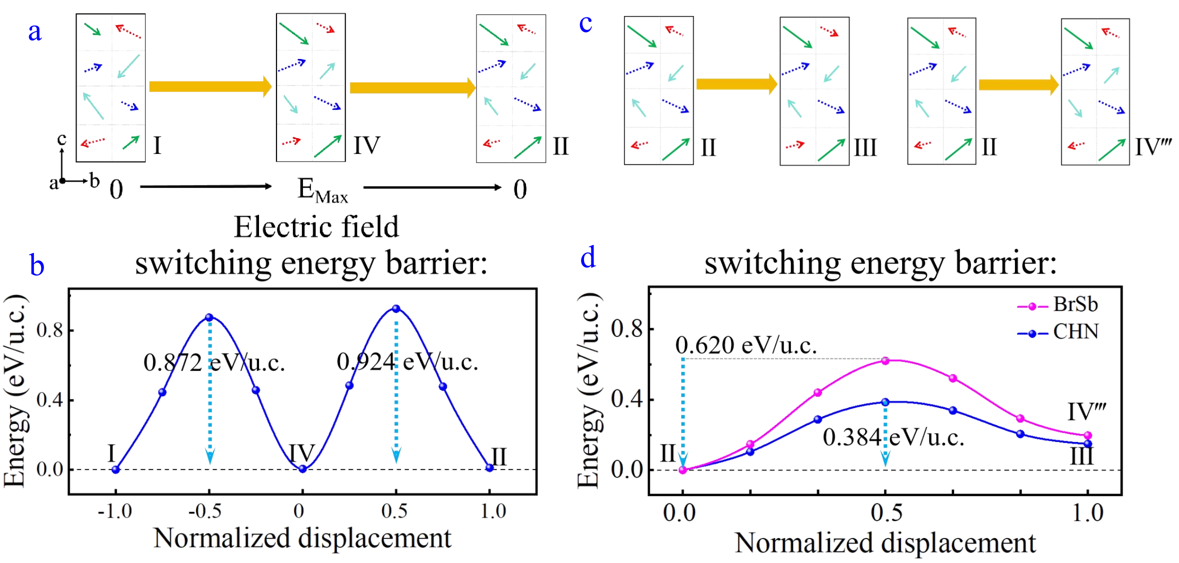

Fig. S11. Possible polarization reversal pathways and the corresponding energy barriers. a, Schematic illustration of a possible (different from the one in the main text) polarization reversal pathway of (MV)[$SbBr_5$] under an electric field: $FiE^{(-)}$ → FE → $FiE^{(+)}$ (I → IV → II), and (b) the corresponding energy barriers. c, Schematic diagrams illustrating the dipole reversal contributed by the inorganic frameworks and the movement of MV cations during the $FiE^{(+)}$ → FE (II → IV); process. d, The dipole reversal energy barriers of the inorganic framework (purple line: II → IV'''') and the MV cations (blue line: II → III) during the $FiE^{(-)}$ → FE process.

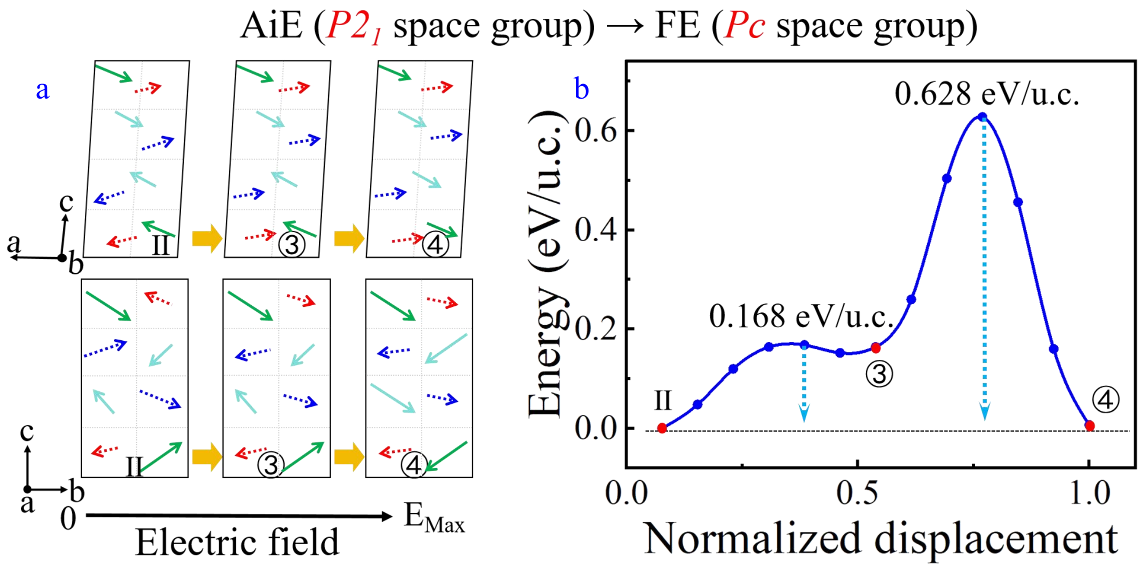

Fig. S12. Another polarization reversal pathway and the corresponding energy barriers. a, The polarization reversal path under an electric field applied along the a-axis is driven by the rotation of dipoles, which induces polarization along both the a-axis and the c-axis, and (b) the corresponding energy barriers.

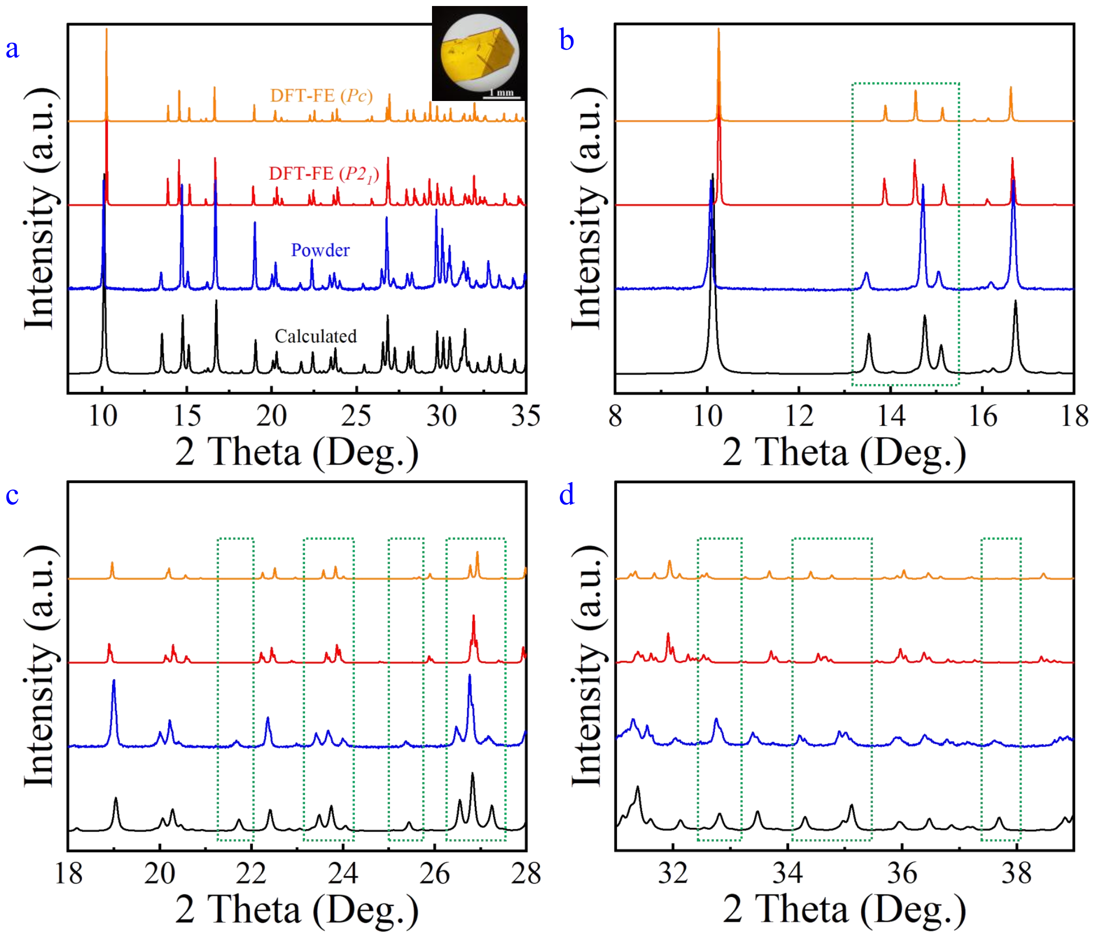

Fig. S13. Further analysis of the (MV)[$SbBr_5$] crystal phase purity. a, Comparison of the calculated XRD pattern for the experimental structure of (MV)[$SbBr_5$], the experimental powder XRD, and the calculated XRD patterns for the two ferroelectric phases from DFT. Inset: Image of the crystal surface observed under a polarized light microscope, showing two distinct regions with single contrast, indicating that both regions are of a single composition. b, c, d, The enlarged detail of the XRD patterns, indicating the high purity of the (MV)[$SbBr_5$] crystal.

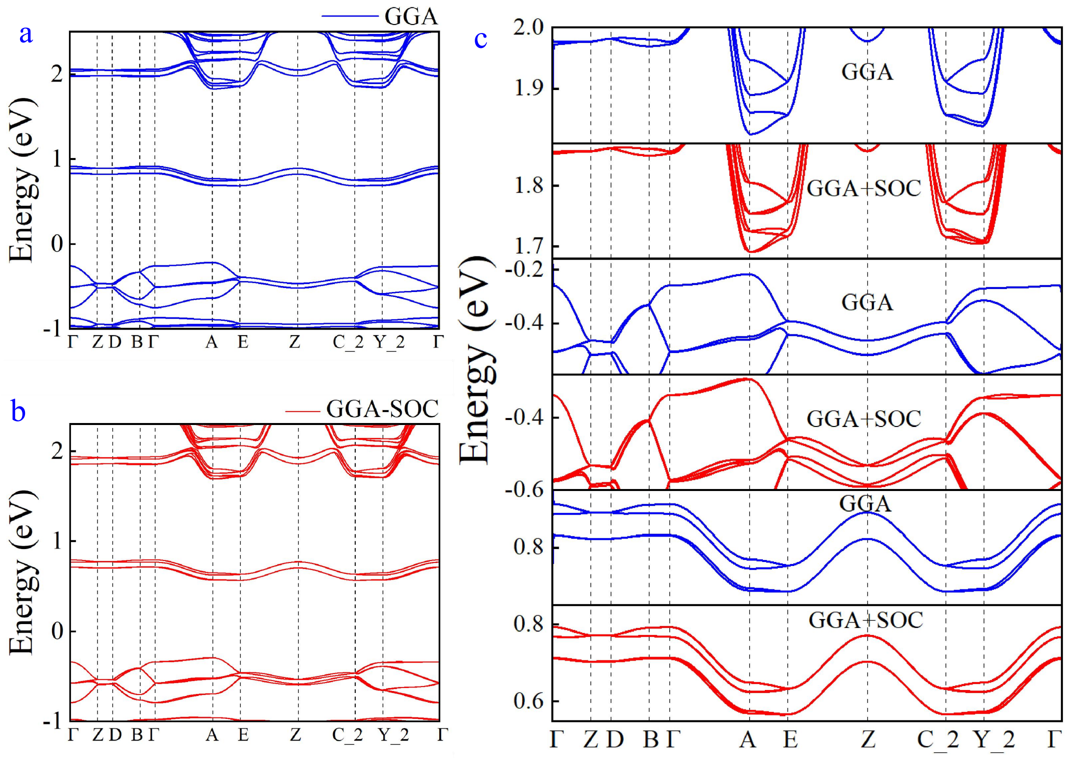

Fig. S14. DFT calculated electronic band structures of (MV)[$SbBr_5$]. a, DFT-GGA + SOC band structure; b, DFT-GGA band structure; c, Local zoom-in of the DFT-GGA + SOC band structure and the DFT-GGA band structure.

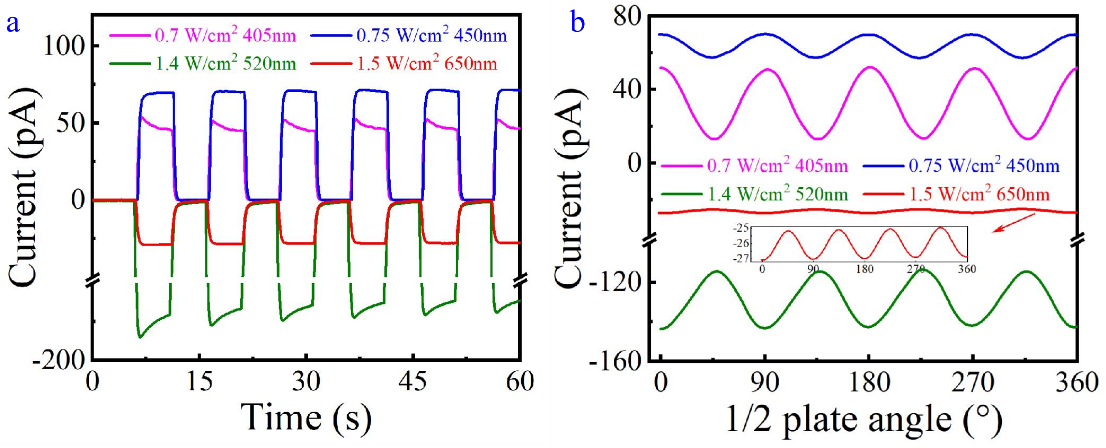

Fig. S15. Photovoltaic responses of (MV)[$SbBr_5$] at different wavelengths. a, I-t responses upon illumination with lights of different wavelengths. b, Room temperature photovoltaic currents under light of different wavelengths vs. λ/2 plate rotation angle.

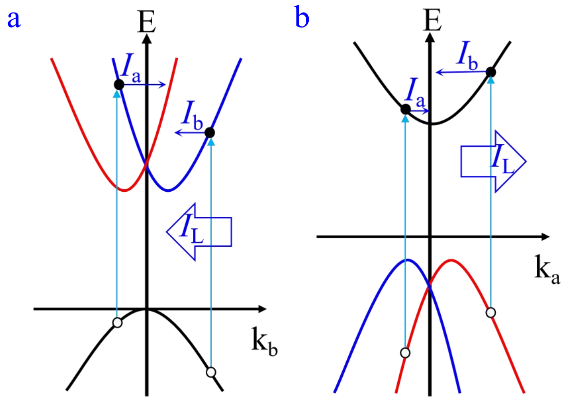

Fig. S16. The influence of valence band and conduction band spin splitting on CPGE photocurrents. a, Under LCP illumination, the spin splitting along the b-axis generates a CPGE photocurrent in the opposite direction along the b-axis; b, Under LCP illumination, the spin splitting along the a-axis generates a CPGE photocurrent in the direction of the a-axis.

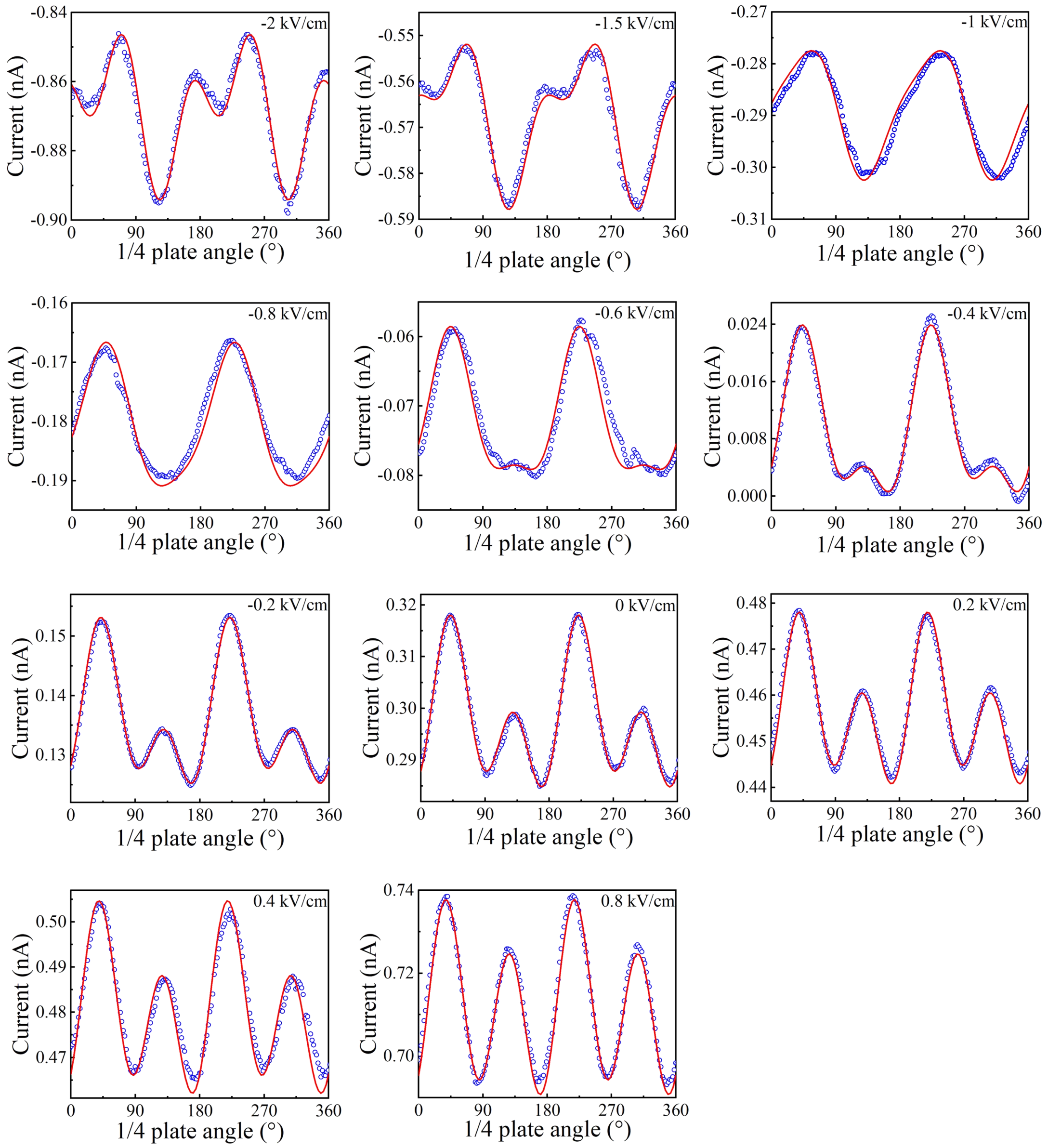

Fig. S17. Circular photogalvanic effect at different bias and the corresponding fitting curve. The blue circulars show the dependence of photocurrent on light polarization under different bias upon 405 nm light illumination. The red line represents the curve fitted according to Eq. 1.

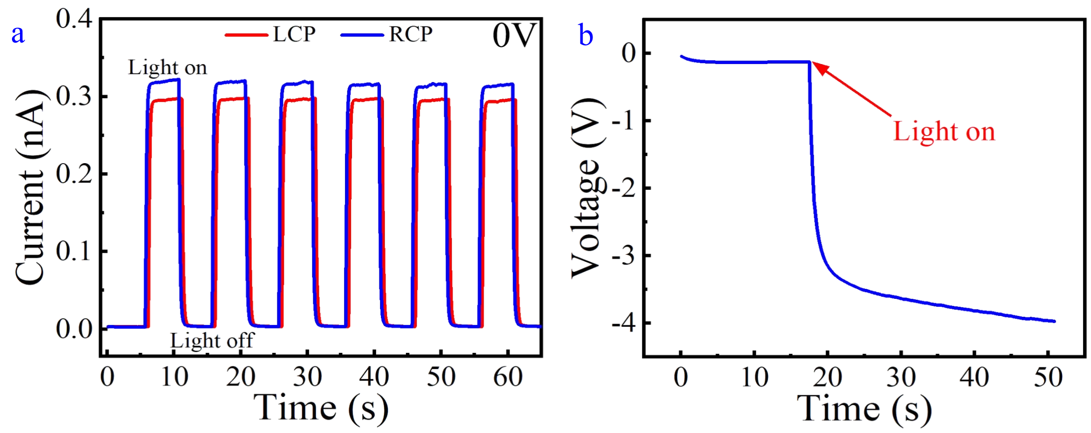

Fig. S18. Photovoltaic responses of (MV)[$SbBr_5$] under circularly polarized lights. a, Photocurrents upon left- (LCP) and right-circularly polarized (RCP) light irradiation at 405 nm with an energy density of 0.7 W/cm^2; b, Open-circuit voltage (V_{oc}) versus time when the light is turned on.

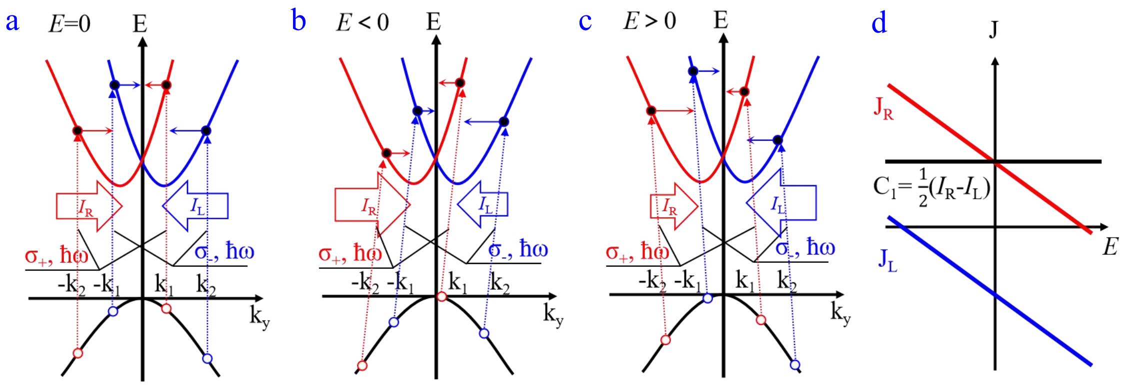

Fig. S19. The effect of bias on the distribution of excited electrons and the CPGE photocurrent. a, The microscopic origin of CPGE arises from the spin splitting of electrons/holes. The RCP/LCP light excitation is represented by dashed lines with red/blue arrows, while the relaxation of the excited electrons is indicated by solid lines with red and blue arrows. Under excitation by RCP (LCP) light at a frequency of ω, energy and momentum conservation only allow transitions at two k_y values labeled $-k_2$ and k_1 ($-k_1$ and k_2). The asymmetric distribution of electrons in k-space results in a non-zero group velocity of the electrons, generating photocurrent I_R (I_L). b and c, The effect of external bias on CPGE. The external bias provides additional momentum to the photoexcited electrons, thereby altering the photocurrent generated under right/left circularly polarized light excitation. However, since the CPGE coefficient $C_0 = (I_R - I_L)/2$, the value of C_1 should remain constant. d, The correlation between the CPGE currents I_R and I_L and the CPGE coefficient C_1 under external bias.

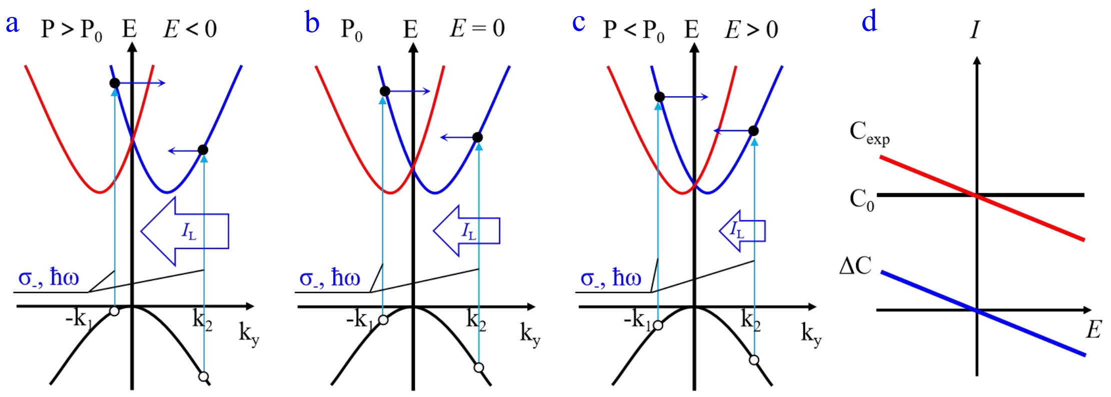

Fig. S20. The effect of bias on polarization, band spin splitting, and CPGE photocurrent. a, Applying a bias along the polarization direction increases the polarization value, enhances the spin splitting of the energy bands, and increases I_L. b, At zero bias, the polarization value is denoted as P_0. c, Applying a bias opposite to the polarization direction decreases the polarization value, reduces the spin splitting of the energy bands, and decreases I_L. d, The experimentally expected CPGE coefficient C_{exp} due to the effect of external bias on polarization, and its relationship with the initial CPGE coefficient C_0, correlates with the compensation photocurrent ΔC.

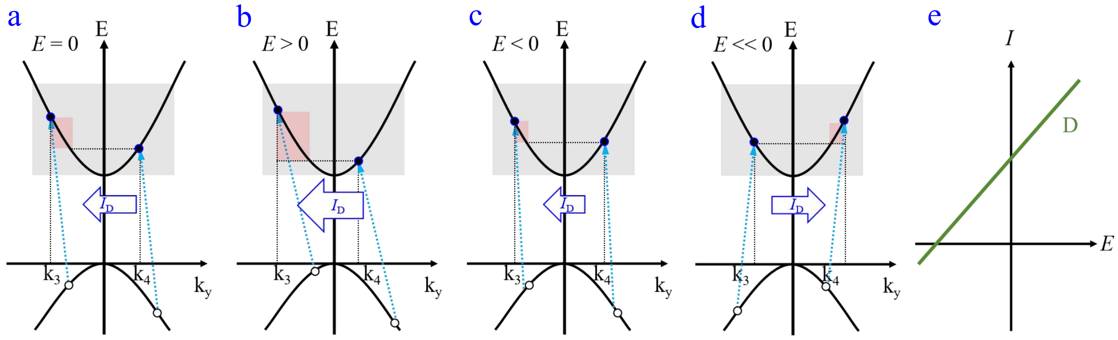

Fig. S21. The effect of bias on the distribution of excited electrons and the LPDE photocurrent. At (a) E=0 , (b) E>0, (c) E<0, and (d) E<<0, explore the influence of bias on LPDE photocurrent. e, The variation in LPDE photocurrent with bias.

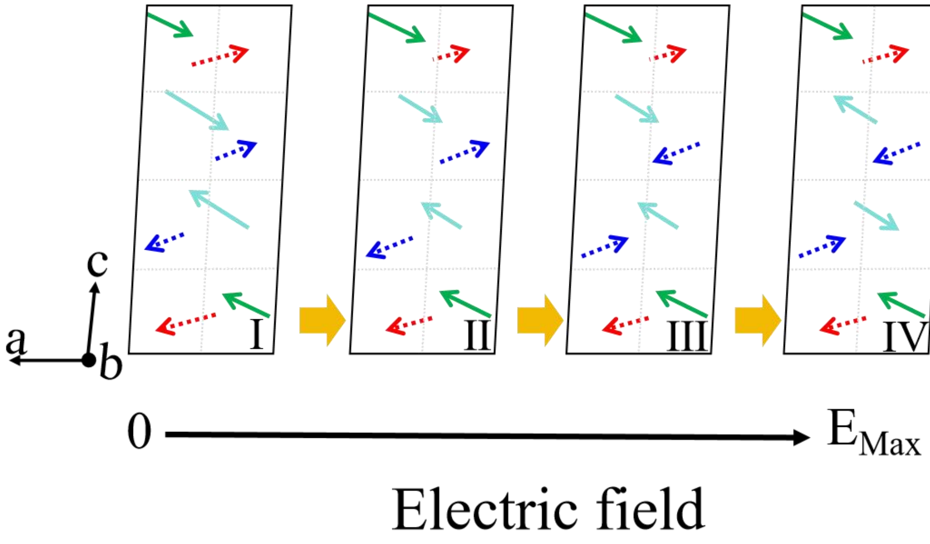

Fig. S22. The dipole evolution in the *ac*-plane under an electric field applied along the *b*-axis shows that the local dipole moment components along the *a*-axis and *c*-axis are consistently canceled by oppositely oriented dipole moments.

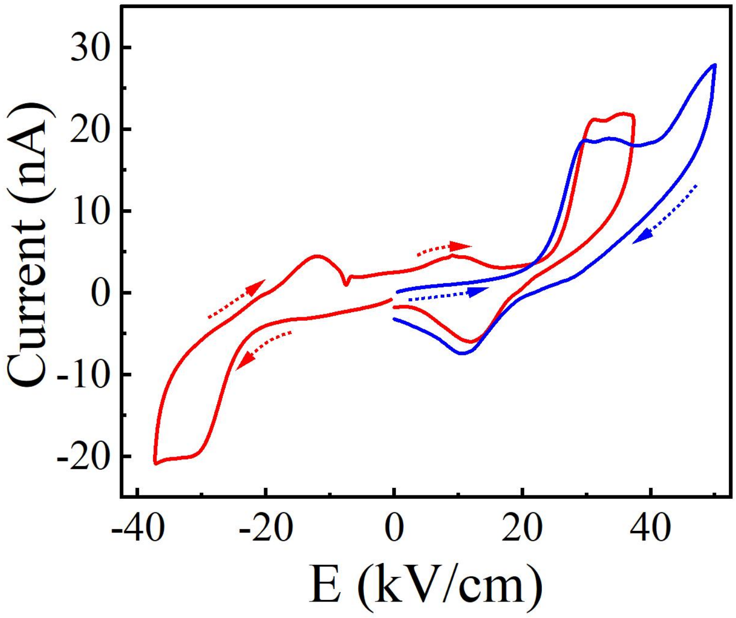

Fig. S23. The current-electric field curve obtained using the Keithley 2636B source meter.

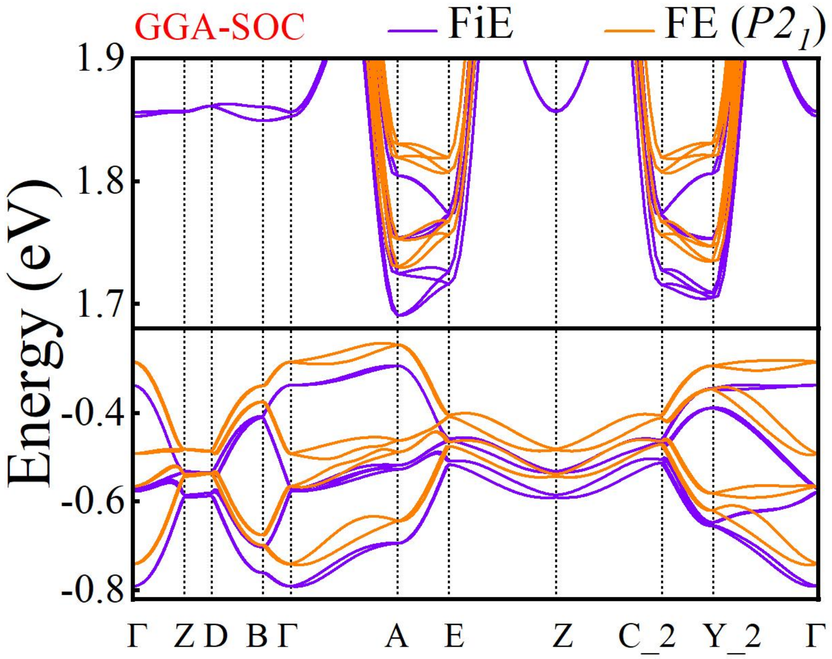

Fig. S24. Comparison of the bands between the ground state FiE and the electric-field-induced ferroelectric state (*P2₁* phase) considering SOC.

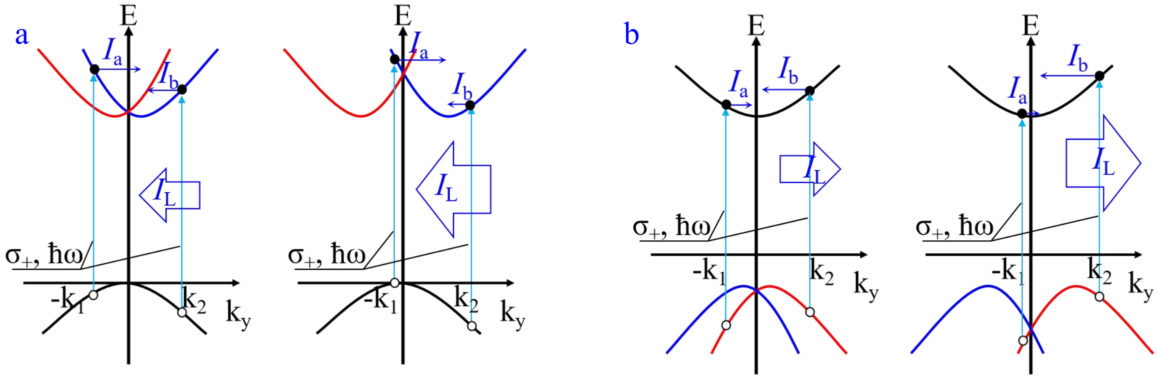

Fig. S25. a and b, show a comparison of the CPGE photocurrent in the ground state FiE and the electric-field-induced ferroelectric state FE, driven by spin splitting dominated by the conduction band and the valence band, respectively, under LCP light excitation.

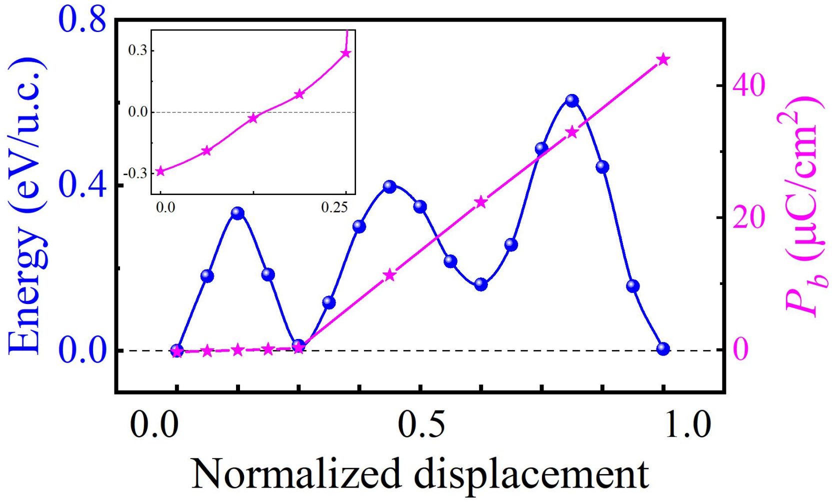

Fig. S26. The calculated polarization as a function of the switching path.

References

1. Kresse G and Furthmüller J. Efficient iterative schemes for *ab initio* total-energy calculations using a plane-wave basis set. *Phys. Rev. B* **54**, 11169-11186 (1996).

2. Perdew JP, Burke K, Ernzerhof M, Generalized Gradient Approximation Made Simple. *Phys. Rev. Lett.* **77**, 3865-3868 (1996).

3. Perdew JP, Ruzsinszky A and Csonka GI *et al.*, Restoring the density-gradient expansion for exchange in solids and surfaces. *Phys. Rev. Lett.* **100**, 136406 (2008).

4. Grimme S, Antony J and Ehrlich S *et al.* A consistent and accurate *ab initio* parametrization of density functional dispersion correction (DFT-D) for the 94 elements H-Pu. *J. Chem. Phys.* **132**, 154104 (2010).

5. Heyd J, Scuseria GE and Ernzerhof M, Hybrid functionals based on a screened Coulomb potential. *J. Chem. Phys.* **118**, 8207–8215 (2003).

6. Mills G, Jónsson H and Schenter GK, Reversible work transition state theory: application to dissociative adsorption of hydrogen. *Surf. Sci.* **324**, 305-337 (1995).

7. King-Smith RD and Vanderbilt D, Theory of polarization of crystalline solids. *Phys. Rev. B* **47**,1651 (1993).

8. Dyakonov MI, Basics of semiconductor and spin physics. Spin physics in semiconductors, 1-37 (2017).

9. McIver J, Hsieh D and Steinberg H *et al.* Control over topological insulator photocurrents with light polarization. *Nature nanotechnology* **7**, 96-100 (2012).
